# Multifunctional Roles of Chlorogenic Acid in Food Packaging Films: Linking Structural Modulation with Active and Intelligent Performance

**DOI:** 10.3390/foods15101637

**Published:** 2026-05-08

**Authors:** Hamid Rajabi, Wanli Zhang, Di Wu, Pang Bo, Young Hoon Jung, Saroat Rawdkuen

**Affiliations:** 1 Unit of Innovative Food Packaging and Biomaterials, School of Agro-Industry, Mae Fah Luang University, Chiang Rai 57100, Thailand; hk.rajabi@yahoo.com; 2 School of Food Science and Engineering, Hainan University, Haikou 570228, China; zwl@hainanu.edu.cn; 3 College of Agriculture & Biotechnology, Zhejiang University, Hangzhou 310058, China; di_wu@zju.edu.cn; 4 Department of Food Science & Technology, Faculty of Science, National University of Singapore, Singapore 117542, Singapore; bopang@nus.edu.sg; 5 School of Food Science and Biotechnology, Kyungpook National University, Daegu 41566, Republic of Korea; younghoonjung@knu.ac.kr

**Keywords:** chlorogenic acid, food preservation, intelligent packaging, biopolymer films, stimuli-responsive systems, controlled release

## Abstract

The transition toward sustainable food packaging requires the integration of biodegradable materials with functional bioactivity. Chlorogenic acid (CGA), a naturally abundant polyphenol, has emerged as a multifunctional compound with the capacity to simultaneously modulate polymer structure and impart active and intelligent functionalities. This review critically examines recent advances in CGA-containing packaging systems, covering fabrication strategies from physical incorporation and chemical grafting to nanostructured and stimuli-responsive architectures. The analysis reveals that CGA plays a dual role. At the molecular level, it regulates the polymer network structure through hydrogen bonding, covalent interactions, and conformational rearrangement. This, in turn, influences mechanical strength, barrier performance, and optical properties. Functionally, CGA provides antioxidant and antimicrobial activity, although its effectiveness depends strongly on the incorporation strategy and concentration. Notably, nanostructured systems and conjugation approaches enable controlled release and enhanced stability. These methods overcome limitations associated with rapid diffusion and environmental degradation, including oxidation, UV exposure, and pH-related instability. Despite these advances, key challenges remain, including CGA instability, uncontrolled release behavior, and limited regulatory and scalability data. Furthermore, while CGA is well established in active packaging, its application in intelligent systems remains limited in the literature, with only a few studies reported on its intelligent applications. Overall, this review highlights the structure–function relationships governing CGA-containing packaging systems and outlines future directions for the rational design of cost-effective, scalable, and multifunctional packaging systems, positioning CGA as a promising component in sustainable strategies for food preservation and waste reduction.

## 1. Introduction

The global food packaging sector is undergoing a fundamental transformation driven by two interconnected challenges: the environmental burden associated with persistent petroleum-based plastics and the significant loss of food across supply chains [1,2]. Conventional plastics are effective as passive barriers against gases, moisture, and external contaminants. However, they are non-biodegradable and contribute to long-term ecological accumulation [3].

At the same time, inadequate packaging performance—particularly the lack of active functionality—accelerates food spoilage, contributing to nearly one-third of global food loss. These limitations highlight the urgent need for packaging systems that are not only environmentally sustainable but also functionally capable of extending food shelf life.

In response, research has increasingly shifted toward active and intelligent food packaging systems. It is crucial to distinguish between these two functional domains. Active packaging is designed to proactively extend shelf life by chemically or biologically interacting with the food or its headspace (e.g., through the controlled release of CGA as an antimicrobial agent). Conversely, intelligent packaging serves as a diagnostic tool. It senses, monitors, and communicates the real-time condition of the food or its environment, such as through CGA-based pH-responsive colorimetric indicators [4]. While active systems focus on preservation and protection, intelligent systems prioritize information and quality tracking. Together, these approaches represent a paradigm shift from passive containment toward multifunctional, responsive packaging technologies.

Central to this transition is the development of renewable and biodegradable polymer matrices, including polysaccharides (e.g., starch, chitosan), proteins (e.g., gelatin, zein), and synthetic biodegradable polymers like polyvinyl alcohol. While these materials offer environmental advantages, their practical application is often limited by poor mechanical strength, high water sensitivity, and a lack of intrinsic bioactivity. Therefore, incorporating naturally derived functional compounds has emerged as a critical strategy to impart antioxidant, antimicrobial, and sensing capabilities [5,6,7].

Among these agents, plant-derived polyphenols have attracted attention due to their strong antioxidant capacity, broad-spectrum antimicrobial activity, and chemical versatility [8,9,10]. Their molecular structures enable not only biological functionality but also direct interaction with polymer matrices through hydrogen bonding and covalent conjugation [11,12]. This dual functionality distinguishes polyphenols from many other bioactive compounds, positioning them as key building blocks for multifunctional packaging.

Chlorogenic acid (CGA) has emerged as a particularly promising candidate within this class. As a naturally abundant ester of caffeic acid and quinic acid, CGA is widely available in agro-industrial by-products such as coffee residues and sunflower seed meals [13,14,15]. Beyond availability, CGA exhibits a unique combination of physicochemical properties, including strong radical scavenging activity and metal-chelating ability [16,17]. Remarkably, its multidentate structure allows it to act as both a bioactive compound and a structural modifier within polymer networks.

Parallel to active packaging, there is a shift toward systems designed to monitor food safety in real time. In this context, CGA offers capabilities that extend beyond traditional preservation. Specifically, CGA-based systems can exhibit pH-responsive color changes and fluorescence-based sensing of spoilage-related compounds, such as biogenic amines. These properties enable the development of optical indicators that provide real-time information on food freshness [18,19]. Consequently, CGA-based systems can bridge the gap between active preservation and intelligent monitoring.

Despite these advantages, several challenges limit the practical application of CGA. Its susceptibility to environmental stressors—such as light and heat—along with uncontrolled release behavior, constrain its effectiveness. To address these limitations, encapsulation has emerged as a promising strategy. This approach enables the preservation of CGA bioactivity and the modulation of release kinetics [20,21]. For instance, Xu et al. [21] developed a chitosan-based composite film incorporating CGA–chitosan oligosaccharide nanoparticles. This system exhibited enhanced barrier properties and effectively extended the shelf life of *Pleurotus geesteranus*, highlighting the potential of nanostructured delivery systems.

Although previous reviews have examined polyphenols in packaging [20,22,23,24,25], CGA has typically been treated as a minor component. A systematic understanding of how CGA simultaneously modulates structural and responsive properties is still lacking. Therefore, this review aims to provide a critical evaluation of CGA-incorporated films. Specifically, it (i) analyzes fabrication strategies ranging from physical incorporation to stimuli-responsive systems; (ii) elucidates how CGA influences the mechanical and barrier properties of different matrices; and (iii) identifies current limitations and research directions required to translate laboratory developments into scalable solutions.

## 2. Methodology

### 2.1. Study Design

This study employed a systematic literature review methodology to critically evaluate the multifunctional roles of CGA in food packaging systems. The review was conducted in accordance with the PRISMA (Preferred Reporting Items for Systematic Reviews and Meta-Analyses) guidelines to ensure transparency, reproducibility, and methodological rigor.

### 2.2. Literature Search Strategy

A comprehensive and systematic search was performed across major scientific databases, including Web of Science, Scopus, and PubMed, to identify relevant peer-reviewed publications. The search covered studies published between January 2015 and early 2026.

The following Boolean search string was applied:

(“chlorogenic acid” OR “5-O-caffeoylquinic acid” OR CGA) AND (“food packaging” OR “active packaging” OR “intelligent packaging” OR “edible film*” OR “nanofiber*”) AND (“antioxidant” OR “antimicrobial” OR “pH-responsive” OR “mechanical property*” OR “barrier property*”).

To enhance sensitivity, database-specific adjustments and truncation symbols were applied where necessary.

### 2.3. Study Selection Criteria

All retrieved records were imported into a reference management system, and duplicate entries were removed prior to screening. The study selection process consisted of two sequential phases: (i) title and abstract screening, and (ii) full-text eligibility assessment.

#### 2.3.1. Inclusion Criteria

Studies were included if they met the following conditions:

- Original peer-reviewed research articles focusing on CGA-incorporated food packaging systems;
- Studies investigating structure–property or structure–function relationships;
- Articles reporting quantitative data on mechanical, barrier, antioxidant, antimicrobial, or functional properties;
- Publications written in English.

#### 2.3.2. Exclusion Criteria

Studies were excluded based on the following criteria:

- Works in which CGA was solely used as a nutritional or pharmaceutical compound without packaging relevance;
- Review articles, book chapters, and conference proceedings (unless providing essential contextual information);
- Studies lacking sufficient experimental or characterization data;
- Non-English publications.

### 2.4. Data Extraction and Standardization

Data were independently extracted from all eligible studies using a predefined standardized extraction matrix. Extracted variables included:

- Biopolymeric matrix composition (e.g., starch, chitosan, polylactic acid);
- CGA incorporation form (free, encapsulated, grafted, or nanostructured);
- Processing/fabrication techniques (e.g., solvent casting, electrospinning, extrusion);
- Functional performance indicators, including tensile strength, water vapor permeability (WVP), DPPH radical scavenging activity, and antimicrobial inhibition zones.

To ensure consistency, extracted data were cross-checked and harmonized prior to analysis.

### 2.5. Quality Assessment and Analytical Framework

The methodological quality and relevance of included studies were evaluated based on predefined criteria, including experimental design robustness, completeness of characterization, and reproducibility of reported results. Although no formal risk-of-bias scoring tool was applied, studies were critically appraised to ensure reliability of the synthesized findings.

### 2.6. Data Synthesis and Categorization

A qualitative and comparative synthesis approach was employed to integrate findings across studies. The literature was systematically categorized into three principal domains based on the structure–function paradigm:

1. Structural modulation: Interaction mechanisms between CGA and polymeric matrices influencing mechanical, thermal, and structural properties.
2. Active functional performance: Evaluation of antioxidant and antimicrobial efficacy across different CGA delivery systems and incorporation strategies.
3. Intelligent functionality: Assessment of CGA-based stimuli-responsive behaviors, particularly pH-sensitive and colorimetric responses in smart packaging applications.

### 2.7. Comparative Analysis

A comparative assessment was performed to identify the most effective CGA incorporation strategies in terms of multifunctional performance. Synthesized results were systematically summarized in Tables 2 and 3, highlighting trends in material performance, functional enhancement, and application suitability. This integrative analysis provides a foundation for identifying research gaps and guiding future developments in bioactive food packaging systems.

## 3. Sustainable Sources, Physicochemical, and Biological Profile of CGA

### 3.1. Sources and Sustainable Recovery of CGA from Agro-Food Residues

CGA, an ester of caffeic and quinic acids, is one of the most abundant polyphenols in plant-based systems and is traditionally sourced from green coffee beans. However, the transition toward a circular bioeconomy has shifted attention from primary agricultural products to agro-food residues as low-cost, renewable, and sustainable CGA sources. These residues—including coffee by-products, oilseed meals, fruit and vegetable wastes, and processing discards—represent structurally diverse matrices in which CGA is often bound within complex lignocellulosic or protein–polysaccharide networks, directly influencing extraction efficiency.

Among these, coffee-derived wastes are particularly rich in CGA. Coffee solid waste has been identified as a major reservoir with strong potential for antimicrobial and antioxidant applications [26]. More specifically, coffee silverskin—the primary by-product of roasting—has been shown to contain high levels of 5-CQA, which can be efficiently recovered using microwave-assisted extraction (MAE). Under optimized conditions (80 °C, 5 min), MAE yielded higher CGA concentrations than conventional hydroethanolic extraction while drastically reducing processing time [27]. This superior performance is attributed to dielectric heating and rapid cell wall disruption, which enhance mass transfer and solvent penetration.

Oilseed residues such as sunflower seed meal represent another high-yield CGA source. Using a COSMO-RS-guided natural deep eutectic solvent (NADES) system, Wang, Fu [28] screened 600 solvent combinations and achieved CGA extraction yields up to 26.39 mg g^−1^ under microwave-assisted conditions. This study highlights a key structure–process relationship: solvent–solute affinity, governed by hydrogen bonding and polarity matching, becomes the dominant factor once mass transfer limitations are minimized, demonstrating the advantage of rational solvent design over empirical selection.

Vegetable processing wastes further expand the resource base for CGA recovery. Potato sprout waste, a typically discarded by-product, yielded up to 4980.05 μg mL^−1^ of 5-CQA under optimized ultrasound-assisted extraction (UAE) conditions [29], with a threefold increase in total antioxidant capacity compared to non-optimized extracts. Similarly, watermelon rind extracts reached 88% recovery efficiency and up to 94% purity when UAE was coupled with macroporous adsorption resin purification [30], emphasizing the importance of integrating extraction with downstream purification to maximize functional yield.

Comparative analysis of extraction techniques reveals distinct performance regimes. MAE generally outperforms UAE in fiber-rich matrices (e.g., fennel waste), as demonstrated by Basile, De Luca [31], due to its ability to overcome structural resistance through internal heating. In contrast, UAE remains advantageous for thermolabile compounds and scalable processes, although its efficiency is often limited by cavitation-dependent mass transfer. This distinction underscores a key structure–process–yield relationship: matrix composition (e.g., fiber content, porosity) dictates the dominant extraction mechanism and optimal technology.

Advanced green solvent systems further enhance CGA recovery by targeting thermodynamic limitations. In a liquid CO_2_–ethanol–water system, CGA yields reached 25.5 mg g^−1^, increasing to 31.6 mg g^−1^ under ultrasonic irradiation [32]. Notably, the study demonstrated that CGA extraction remains partially diffusion-limited, while other compounds (e.g., rutin) are primarily governed by solubility, highlighting compound-specific extraction behavior. This reinforces that process optimization must consider both mass transfer and equilibrium solubility constraints.

Emerging non-thermal technologies such as high-voltage electric discharge (HVED) provide alternative extraction pathways, although with comparatively lower yields (up to ~3.66 mg/100 g depending on waste fraction) [33]. These methods rely on electrical disruption of plant tissues but remain sensitive to process parameters such as frequency and treatment time, indicating that their efficiency is still limited relative to MAE and optimized solvent systems.

In addition to extraction, analytical and monitoring tools are essential for process optimization. For example, Cuffaro, Palladino [34] developed a rapid and highly precise colorimetric method (R^2^ = 0.9996; recovery ≈ 106%) for CGA detection in artichoke waste, enabling efficient tracking of extraction performance. Furthermore, sweet potato leaves have been identified as a CGA-rich biomass with optimized ethanol-based extraction conditions, achieving a crude extraction yield of 3.54% [35], supporting their potential as a functional ingredient source.

Overall, quantitative benchmarking across studies indicates that CGA yields vary widely—from mg/100 g levels in emerging technologies (HVED) to over 30 mg g^−1^ in optimized green solvent systems—depending on raw material structure, solvent design, and extraction technology. MAE and pressurized green solvent systems generally deliver the highest efficiencies, while UAE offers flexibility and compatibility with downstream purification. Importantly, no single method is universally optimal; instead, the interplay between matrix structure, solvent properties, and energy input defines extraction performance. These findings highlight that sustainable CGA recovery requires an integrated design approach combining green chemistry principles, process intensification, and matrix-specific optimization strategies.

### 3.2. Physicochemical and Biological Profile of CGA

**Figure 1:**
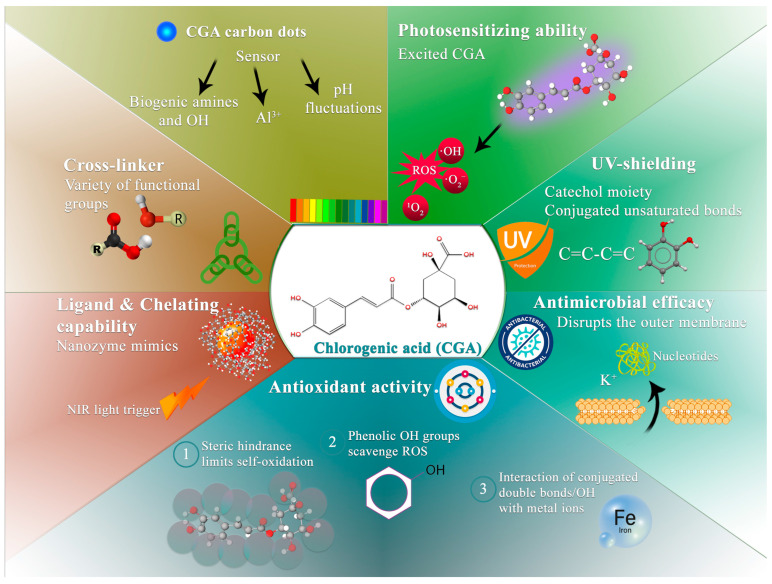
Chemical structure and primary intrinsic bioactivities of CGA relevant to food packaging, including antioxidant mechanisms, antimicrobial disruption, and biosafety.

To effectively engineer active packaging systems, it is essential to first understand the fundamental chemistry of CGA and the specific mechanisms by which it combats food spoilage. The following subsections delineate the structure–function relationships that dictate its behavior within polymer matrices, ultimately determining its functional performance in active packaging systems (Figure 1).

#### 3.2.1. Chemical Structure and Stability Challenges

CGAs comprise a diverse group of phenolic esters biosynthesized via the shikimic acid pathway through the esterification of trans-cinnamic acids with (–)-quinic acid [36]. Among these, 5-O-caffeoylquinic acid (5-CQA) is the most abundant isomer in natural sources, particularly in green coffee beans, and is also widely present in beverages such as coffee and tea. Owing to its prominent biological functionality and prevalence, 5-CQA is commonly referred to as CGA in the international market [37]. A critical physicochemical parameter of CGA is its dissociation constant (pKa ≈ 3.92), which dictates its ionization state and, consequently, its solubility and molecular interactions at varying pH levels.

Despite its potent bioactivity, CGA is highly sensitive to environmental stressors such as high temperature, alkaline pH, and light exposure [38,39,40]. Under these conditions, CGA readily undergoes isomerization (acyl migration) or hydrolysis into caffeic and quinic acids.

Furthermore, the catechol group makes it susceptible to oxidation. This leads to the formation of quinones and subsequent browning, which can impair the visual quality of the film [41]. Such inherent instability underscores the necessity of encapsulation and matrix integration strategies [5,42,43,44,45].

These physicochemical properties also govern CGA–polymer interactions, particularly through hydrogen bonding and electrostatic interactions, which influence its dispersion, stability, and release behavior in packaging matrices [42].

#### 3.2.2. Antibacterial and Antibiofilm Mechanisms

CGA exhibits broad-spectrum antimicrobial activity, primarily targeting foodborne bacteria and spoilage fungi (molds and yeasts), through multiple, complementary mechanisms [46,47]. While some studies have explored its broader pharmacological potential against other microorganisms, its relevance in food packaging systems is most prominently established in inhibiting bacterial and fungal growth. A primary mode of action involves the disruption of the bacterial cell membrane, leading to irreversible increases in permeability and cellular damage [48].

In Gram-negative bacteria, the stability of the outer membrane is partially maintained by divalent cations (e.g., Mg^2+^ and Ca^2+^) that electrostatically bridge adjacent lipopolysaccharide (LPS) molecules [49,50]. CGA, which possesses a carboxylic acid group and catechol hydroxyl groups, can act as a chelating agent depending on the environmental pH. At physiological or near-neutral pH levels—typical of many food systems—CGA exists primarily in its deprotonated, anionic form, enabling it to sequester these essential cations. This chelation process disrupts the electrostatic stabilization of the LPS layer, thereby weakening membrane integrity and compromising its barrier function [51].

These mechanisms are further elucidated in pathogen-specific studies. For instance, in *Yersinia enterocolitica*, CGA effectively inhibits growth and suppresses biofilm formation—structured microbial communities that often exhibit high resistance to sanitation. Research by Chen, Peng [52] demonstrated that CGA interferes with both biofilm initiation and maturation by inhibiting swimming motility and destabilizing established biofilm architecture. These effects are fundamentally linked to CGA-induced membrane damage, characterized by the leakage of intracellular constituents and a loss of metabolic viability.

#### 3.2.3. Antifungal Activity and Mechanisms

CGA exhibits broad-spectrum antifungal activity against phytopathogenic molds and spoilage yeasts via a multi-target mode of action. Rather than inducing solely nonspecific structural damage, CGA disrupts key fungal physiological and metabolic processes, ultimately inhibiting growth and reducing viability. This mechanistic versatility is particularly advantageous for active food packaging applications, as it enables sustained antifungal efficacy while minimizing the risk of resistance development.

At the early stages of infection, CGA acts as an effective preventive agent by inhibiting fungal spore germination and germ tube elongation, thereby blocking the transition from dormant spores to invasive mycelia and reducing postharvest decay [53]. At the cellular level, CGA induces endoplasmic reticulum stress, leading to dysregulated Ca^2+^ signaling and mitochondrial Ca^2+^ overload. This cascade activates oxidative stress responses, impairs mitochondrial respiration, and depletes ATP levels, resulting in reduced fungal viability, as specifically demonstrated in *Botrytis cinerea* models [54].

In parallel, CGA compromises fungal membrane integrity by targeting ergosterol biosynthesis. Specifically, CGA suppresses the expression and activity of key sterol biosynthetic enzymes, such as *ERG4* and *ERG6*. This suppression leads to decreased ergosterol content, enhanced lipid peroxidation, and increased membrane permeability [55]. Mitochondrial dysfunction represents a convergent antifungal mechanism across various species. In yeasts, CGA induces membrane depolarization and excessive reactive oxygen species (ROS) production, eventually triggering apoptosis-like cell death [56].

#### 3.2.4. Antioxidant Activity

The molecular structure of CGA is characterized by multiple phenolic hydroxyl groups [57]. This configuration enables the direct scavenging of reactive oxygen species (ROS) through hydrogen atom transfer (HAT), thereby terminating oxidative chain reactions and stabilizing radical species. Furthermore, the steric hindrance associated with the CGA molecule limits self-oxidation, reducing the risk of pro-oxidant effects. Moreover, the combined presence of phenolic hydroxyl groups and conjugated double bonds confers a strong electron-donating capacity. This facilitates efficient electronic interactions with the d-orbitals of metal ions [16]. Consequently, CGA-loaded packaging systems consistently demonstrate pronounced radical-scavenging capacity, contributing to delayed lipid and protein oxidation in food products. Accordingly, CGA-loaded packaging systems have consistently demonstrated pronounced radical-scavenging capacity, contributing to delayed lipid and protein oxidation in packaged foods [55,58].

Beyond direct radical neutralization, CGA plays a critical role in protecting food macromolecules. In protein-rich foods, particularly muscle-based products like fish, CGA intercepts reactive species that would otherwise oxidize sulfhydryl groups (–SH) into disulfide bonds (–S–S–). This interception prevents protein aggregation and the subsequent loss of quality [58].

## 4. Active and Intelligent Films Containing CGA

### 4.1. Biopolymer-Based Films

The first generation of CGA-incorporated films involve the integration of the bioactive agent into continuous biopolymer matrices, primarily via the solvent casting method. In these systems, CGA plays a dual role: it functions as a releasable active agent and a structural modifier. The efficacy of these films is governed by molecular interactions—specifically hydrogen bonding and covalent grafting—between the phenolic hydroxyl groups of CGA and the polymer chains. These interactions significantly alter the film’s physicochemical properties. In Table 1, systems are categorized as either self-standing films or in situ coatings, depending on their application mode.

#### 4.1.1. Polysaccharide-Based Matrices

Polysaccharide-based biopolymers, including starch, chitosan, and cellulose, are the most widely investigated matrices for CGA-containing packaging. While they offer renewability, their intrinsic hydrophilicity and mechanical fragility often restrict direct application. Within this context, CGA emerges as a multifunctional modifier that regulates the polymer network structure via its phenolic hydroxyl and carboxyl moieties.

In starch-based films, CGA functions primarily as a molecular crosslinker. The dense hydrogen bonding formed between CGA and amylose/amylopectin chains restricts polymer chain mobility. As reported by Zhang, Zhang [59], these interactions reduce the free volume available for water diffusion. This leads to lower water vapor permeability and enhanced dimensional stability. The synergistic action of CGA-mediated crosslinking and physical densification yields films with improved integrity. Simultaneously, the unsaturated bonds and aromatic rings of CGA confer strong UV-shielding capacity. Recently, Li et al. [60] developed starch-based films crosslinked with citric acid and incorporated with CGA. The optimized formulation (4% citric acid and 15% CGA) exhibited strong bioactivity, with a minimum inhibitory concentration (MIC) of 12.5 mg/mL against *Staphylococcus aureus*, alongside a 91% DPPH• radical scavenging activity.

**Table 1:** Applications of CGA in biopolymer-based films and surface coatings for food packaging.

Packaging System Category	Polymer Matrix/Architecture	CGA Incorporation Strategy & Dominant Interactions	Primary Functional Outcomes	Key Limitations and Bottlenecks	Ref.
I. Biopolymer matrices	Chitosan (CH)	Physical blending (hydrogen bonding); covalent grafting via formation of amide band (between NH_2_ of CH and –COOH of CGA) or esterification (between –OH of CH and –COOH of CGA)	Enhanced antioxidant and antimicrobial activity; improved oxygen barrier; tunable release via conjugation	Reduced mechanical properties at high grafting density; processing complexity	[61]
	Starch	Physical entrapment via hydrogen bonding between functional groups of CGA and starch –OH; CGA acts as a cross-linking agent	Improved UV-shielding, antioxidant, and antimicrobial activity; enhanced mechanical properties	-	[59]
	Agar	Physical entrapment via hydrogen bonding	Reinforced tensile strength; enhanced barrier performance	Reduced flexibility at high CGA-loading	[62]
	Cellulose/nanocellulose	Surface adsorption through hydrogen, C–O, and C–OH bonds	Enhanced mechanical properties at low CGA-loading; improved antioxidant and antimicrobial activity and hydrophobicity	Tradeoff between functional properties and mechanical properties at high CGA-loading; lack of inhibitory activity over *Penicillium* spp. infection	[63]
	Zein	Physical entrapment via hydrogen bond and electrostatic interaction between zein and CGA	Enhanced mechanical properties and antioxidant and antimicrobial activity	Reduction thermal stability	[64]
II. Nanostructured and engineered systems	Nanotube-based composites	Encapsulation of CGA within nanotube lumen via hydrogen bonding and hydrogen bonds between nanotube and fibers	Suppressed burst release; prolonged antioxidant and antimicrobial efficacy; tunable release	Reduced mechanical properties and barrier performance	[65]
III. Intelligent and stimuli-responsive architectures	Metal–phenolic networks	Hydrogen bonds and hydrophobic forces between matrix and Cu-CGA nanoparticles; coordination bonding between CGA and metal ions	NIR-triggered photothermal sterilization; synergistic antimicrobial action; enhanced tensile strength and antioxidant capacity	Reduced flexibility; Potential metal ion migration; regulatory considerations	[66]
	Cascade nanoreactors	Hydrogen bonds between CaO_2_@CGAFe nanoparticles and matrix	continuously release H_2_O_2_; Self-activated in situ ROS generation; dual-mode antibacterial/antifungal behavior of “on-demand burst and routine protection”; NIR-triggered photothermal sterilization	Safety evaluation of ROS exposure; system complexity	[16]
	Fluorescent sensing platforms (CGA-derived carbon dots)	AFM topography and SEM micrographs, and UV light confirmed doping carbon dots into matrix.	Fluorescence of the CGA-derived carbon dots is both excitation- and pH-dependent; film possessed good resistance to photobleaching and to detect Al^3+^ residues in food, and to sense basic substances.	Limited industrial validation; sensor calibration requirements	[18]

In chitosan systems, the dominant role of CGA shifts toward oxidative stabilization. Since chitosan is inherently antimicrobial, the physical addition of CGA does not always substantially enhance microbial inhibition. Instead, as demonstrated by Cao, Islam [58], CGA significantly improves oxygen barrier properties and suppresses lipid/protein oxidation. This suggests that CGA functions primarily as an antioxidant stabilizer in bioactive polysaccharides.

However, recent research indicates that the method of incorporation—physical blending versus chemical conjugation—drastically alters performance. Hu, Sun [61] compared the effects of grafting CGA onto the chitosan backbone versus simple physical incorporation. They found that conjugated films exhibited superior bioactivity and preservative efficacy. This suggests that covalent attachment provides a more stable delivery mechanism than physical entrapment, effectively enhancing both antioxidant and antibacterial properties.

Finally, native cellulose often lacks sufficient mechanical robustness for direct packaging applications [67,68]. One effective approach involves incorporating bioactive polyphenols to improve both functional performance and water resistance. For instance, nanocellulose–starch composite films containing polyphenolic additives showed moderate improvements in structural stability [69]. More specifically, Krysa, Szymańska-Chargot [63] showed that CGA adsorbs onto micro- and nanofibrillated cellulose surfaces through hydrogen bonding and hydrophobic interactions. This surface modification strategy enhanced antioxidant functionality without compromising tensile strength, addressing a common trade-off in cellulose-based composites.

#### 4.1.2. Protein-Based Matrices

Proteins possess excellent film-forming ability and abundant reactive side chains. These side chains provide specific binding sites for polyphenols, distinguishing protein matrices fundamentally from neutral polysaccharides. Accordingly, the incorporation of CGA into protein-based matrices is governed primarily by two interconnected mechanisms: the conformational rearrangement of protein secondary structures and covalent or non-covalent crosslinking.

Unlike polysaccharides, proteins exhibit hierarchical secondary and tertiary structures that are highly sensitive to polyphenol interactions. Chen, Liu [70] demonstrated that CGA functions as a structural modulator in gelatin/wheat gliadin electrospun films. Its incorporation induced a transition from α-helix–dominated conformations to β-sheet structures. This structural rearrangement reflects a “tightening” of the protein network. This process is driven by hydrogen bonding and hydrophobic interactions between CGA and amino acid residues. As a result, the protein chains become more compactly organized. This yields films with enhanced thermal stability, which is an essential attribute for packaging materials exposed to thermal processing or fluctuating storage conditions.

Beyond non-covalent interactions, CGA has also been effectively integrated into protein matrices through covalent conjugation. Fu, Wu [71] synthesized CGA–gelatin conjugates as active coatings for fresh seafood preservation. They confirmed the successful grafting of CGA onto the gelatin backbone, identifying an optimal CGA-to-gelatin molar ratio of 4:1. Importantly, these conjugates exhibited superior antioxidant performance compared with free CGA, while the antibacterial activity was fully retained. This enhancement was attributed to the stabilized phenolic structure and improved dispersion achieved through covalent attachment. This demonstrates that conjugation can amplify CGA functionality in protein-based systems.

In response to sustainability concerns associated with animal-derived proteins, recent studies have increasingly focused on plant protein matrices. Wang, Xu [7] investigated films based on walnut protein isolate (WNPI) complexed with propylene glycol alginate (PGA) and incorporated CGA. The resulting WNPI/PGA–CGA films exhibited simultaneous antioxidant and antibacterial activity. This confirms that plant proteins can effectively host CGA through interfacial interactions while delivering multifunctional preservation performance.

#### 4.1.3. Hybrid Protein–Polysaccharide Systems

Hybrid matrices combine proteins and polysaccharides to overcome the intrinsic limitations of single-component biopolymers. These systems integrate complementary mechanical strength, barrier performance, and bioactivity. Within these architectures, CGA functions not merely as a releasable compound but as a critical interfacial modifier. It improves phase compatibility and reinforces the composite network.

In sweet potato starch/sweet whey protein hybrid matrices, CGA acts as a molecular bridge. Its multiple hydroxyl and carboxyl groups facilitate extensive hydrogen bonding with both starch chains and whey protein molecules [55]. This interfacial crosslinking significantly enhances film toughness and swelling behavior. At a CGA loading of 3%, the films exhibit synergistic improvements in barrier properties and UV-shielding performance.

Building on this approach, Shuang, Zong [72] incorporated CGA into silk fibroin (SF) films reinforced with cellulose nanocrystals (CNCs). In this multicomponent matrix, CGA induced a conformational transition in the protein phase, promoting β-sheet formation within the SF network. This structural reorganization, combined with dense hydrogen bonding between SF and CNCs, resulted in increased surface hydrophobicity and reduced water vapor permeability. Beyond structural enhancement, the hybrid films exhibited pronounced antibacterial activity and effectively inhibited fungal spore germination.

### 4.2. Nanostructured Systems

The direct incorporation of CGA into polymeric films often results in uncontrolled release kinetics. Furthermore, CGA is sensitive to heat, light, and pH fluctuations, which can lead to degradation during processing. Nanostructured systems overcome these limitations by confining CGA within nanoscale architectures. This enables spatial control, molecular protection, and programmable release behavior. Among these strategies, electrospinning and nanoencapsulation are particularly effective tools for tailoring the long-term efficacy of CGA in active packaging.

#### 4.2.1. Electrospun Nanofibers

Electrospinning enables the fabrication of nonwoven fibrous mats with high surface-area-to-volume ratios and interconnected porosity. These characteristics make them ideal as active coatings or functional interlayers. Protein-based polymers are especially attractive electrospinning matrices due to their amphiphilic character and abundant functional groups.

In gelatin–wheat gliadin nanofibers, CGA incorporation modulates fiber morphology and thermal behavior. This indicates strong intermolecular interactions between CGA and protein chains during jet elongation and solvent evaporation [70]. These interactions reinforce the nanofibrous network while enabling a sustained release profile. The resulting controlled diffusion prolongs CGA activity, maintaining antibacterial and antioxidant functionality over extended storage periods.

Similar structure–function relationships have been observed in zein-based electrospun systems. Wang, Pan [41] demonstrated that CGA–zein interactions significantly enhance the mechanical strength of nanofiber films. Instrumental analyses revealed that hydrogen bonding and electrostatic interactions dominate the binding process. This leads to static quenching and the formation of stable complexes. Importantly, these molecular interactions translate directly into macroscopic property changes. They reinforce mechanical integrity while simultaneously imparting bioactivity to coaxial electrospun nanofibers. Collectively, these findings underscore how nanoscale protein–polyphenol interactions can be engineered to balance mechanical performance and biological functionality.

#### 4.2.2. Nanoparticles and Nanoclays

In addition to organic nanofibers, inorganic nanocarriers offer an effective strategy for stabilizing CGA. These carriers regulate CGA release through precise structural design. Dong, Dong [73] employed CGA-functionalized layered double hydroxides (LDHs@CGA) to fabricate a three-layer active film on a biodegradable polyvinyl alcohol (PVA) substrate. A PVA-based active coating containing the functionalized nanoclays was applied via a dual-coating technique, ensuring seamless interfacial integration.

The layered LDH architecture created a tortuous diffusion pathway. This structure effectively controlled CGA release and mitigated the burst-release behavior typical of monolayer films. Consequently, LDHs@CGA-loaded films exhibited high antioxidant (85.6%) and antibacterial (87.2%) activities. Moreover, the uniform dispersion of nanoclays significantly enhanced oxygen barrier properties. This demonstrates that inorganic nanostructures can simultaneously improve barrier performance and extend the functional lifespan of biodegradable active packaging materials.

### 4.3. Stimuli-Responsive Active Packaging Systems

**Figure 2:**
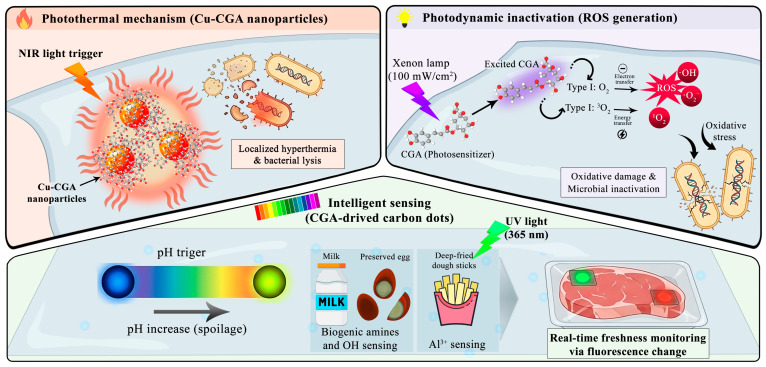
Advanced applications of CGA in smart food packaging, illustrating the operational mechanisms of intelligent spoilage sensors (colorimetric/fluorescent carbon dots) and stimuli-responsive (photothermal and photodynamic) antimicrobial systems.

The third generation of CGA-incorporated films marks a paradigm shift from passive release mechanisms to interactive and responsive systems. In these advanced platforms, CGA is utilized not only as a bioactive additive but also as a functional precursor for nanomaterials capable of responding to specific external stimuli. This enabling technology allows for on-demand preservation, where the packaging remains latent until triggered by environmental or physical factors, thereby optimizing efficacy while minimizing additive interaction with the food (Figure 2).

#### 4.3.1. CGA-Derived Carbon Dots

A groundbreaking development involves the conversion of CGA into carbon dots (CDs), transitioning its role from a simple molecular additive to a precursor for zero-dimensional quantum nanomaterials that retain antioxidant functional groups. Mao, Dong [74] pioneered the integration of these CGA-derived CDs into sustainable alginate films. By synergistically combining CGA-CDs with CGA-functionalized layered clays, they created a nanocomposite architecture where the CDs acted as bioactive fillers that reinforced the polymer network. This integration significantly enhanced UV-blocking capacity, hydrophobicity, and thermal stability compared to neat alginate films, proving that nanoconversion offers a sophisticated route to resolve the mechanical limitations of polysaccharide matrices while amplifying their active protective performance.

#### 4.3.2. Photothermal and Photodynamic Films

Beyond structural reinforcement, the intrinsic chemical reactivity of CGA has been exploited to construct antimicrobial systems activated on demand. Han, Chen [62] addressed the sensory limitations of high CGA concentrations by integrating it with photodynamic inactivation (PDI) technology. In PDI, light-activated agents generate reactive oxygen species (ROS), such as ·OH, ^1^O_2_, and ·O_2_^−^, inducing lethal oxidative stress in pathogens [75,76]. While CGA is not classified as a traditional photosensitizer, its photodynamic potential is a conditional functionality that emerges in specific polymeric or nano-enabled environments. Notably, even in non-metallic systems like agar-based films, CGA exhibits significant photodynamic efficacy when subjected to high-intensity light (e.g., Xenon lamps), likely due to the stabilization of its excited states within the polymer network.

In parallel, chemically responsive platforms based on nanozyme engineering have emerged. Leveraging metal–ligand coordination, peroxidase-like nanozymes constructed via metal–CGA coordination catalyze H_2_O_2_ into reactive hydroxyl radicals (·OH). As reported by Ping, Zhang [16], CGA serves as a chelating ligand in CaO^2^@CGA–Fe nanoparticles, enabling a “substrate self-supply” mechanism for ROS production. Upon near-infrared (NIR) irradiation, localized hyperthermia (up to 56.1 °C) is generated, exponentially increasing catalytic rates. Similarly, Jiang, Sheng [66] developed Cu–CGA nanoparticles where NIR stimulation triggers photothermal conversion (43.6 °C). These systems function as “smart switches,” allowing for precise, spatiotemporal control over antimicrobial activity.

### 4.4. Intelligent Packaging: Sensing and Monitoring Functions

While the aforementioned systems focus on preservation (Active), intelligent packaging utilizes CGA’s unique optical and chemical sensitivity to sense and communicate the real-time condition of the food. These diagnostic tools focus on information transfer and quality monitoring rather than direct microbial intervention.

#### 4.4.1. CGA-Derived CDs as Photoluminescent Probes

The conversion of CGA into carbon nanodots via hydrothermal carbonization preserves its essential functional groups, such as phenolic hydroxyl and carboxylic acid moieties, within a conjugated carbon framework. As demonstrated by Zhang, Wang [18], these CGA-CDs exhibit strong photoluminescence with a high degree of photostability. When integrated into a poly(vinyl alcohol) (PVA) matrix, the resulting films maintain excellent transparency and mechanical integrity while functioning as a fluorescent diagnostic platform. The intelligent nature of these films stems from their ability to undergo predictable optical changes in response to chemical markers of food deterioration.

#### 4.4.2. Spoilage Monitoring: pH and Amine Sensing

The primary intelligent function of CGA-CD-based films is the real-time monitoring of food freshness. During the spoilage of protein-rich foods (e.g., meat and seafood), the accumulation of total volatile basic nitrogen (TVB-N) and other alkaline substances, such as ammonia and biogenic amines, leads to localized pH shifts.

Owing to the pH-sensitive phenolic groups on their surface, the fluorescence intensity of CGA-CDs is highly responsive to these changes. Specifically, the films act as visual or fluorometric indicators; as the internal environment of the package becomes more basic due to microbial metabolism, the optical response of the CGA-CDs changes accordingly [18]. This provides a non-destructive method for consumers and producers to assess the degree of spoilage without opening the package, representing a hallmark of intelligent packaging technology.

#### 4.4.3. Detection of Contaminants and Additives

Beyond freshness monitoring, CGA-based intelligent films serve as sensitive detectors for specific food contaminants and residues. The functional groups on CGA-CDs enable selective interaction with metal ions through chelation or electrostatic mechanisms. Research indicates that these films can effectively sense Al^3+^ ions, which are often found as residues in food products containing aluminum-based additives.

The presence of Al^3+^ triggers a significant fluorescence enhancement or “turn-on” response in the CGA-CDs, likely due to the inhibition of photoinduced electron transfer or the enhancement of structural rigidity upon coordination. By integrating this sensing capability into the packaging material, CGA-based systems offer a dual-purpose solution: they function as active barriers to oxidation and intelligent sensors for food safety, providing a comprehensive strategy for modern food quality management [18].

## 5. Effect of CGA on Physicochemical and Functional Properties of Active Films

### 5.1. Structural Characteristics

#### 5.1.1. Morphological Modifications and Film Topography

The integration of CGA into biopolymer matrices induces pronounced morphological changes. These changes are primarily governed by modifications in solution rheology and the molecular compatibility between CGA and polymer chains. Scanning electron microscopy (SEM) analyses reveal that CGA acts as a structural modifier. It influences fiber diameter in electrospun mats and surface topography in solvent-cast films. The extent of these morphological changes directly correlates with the compatibility between CGA and the film-forming polymers. The effects of CGA on the mechanical and structural properties of biopolymer-based films are summarized in Table 2.

In electrospun systems, CGA incorporation consistently increases nanofiber diameter. This effect is attributed to CGA-induced rheological modifications. The abundant functional groups in CGA form extensive hydrogen bonds with polymer backbones, which significantly increases solution viscosity. This elevated viscosity resists jet elongation under electrostatic forces. Consequently, stretching is limited, yielding thicker fibers [70,77,78]. For example, encapsulating 4% CGA in polyvinylpyrrolidone nearly doubled the fiber diameter from 258.7 nm to 494.0 nm [77]. At higher loadings, fiber singularity may be compromised. Excessive CGA concentrations promote fiber adhesion and merging at contact points [65,70]. This behavior likely arises from uneven solvent evaporation and disrupted jet fluency, shifting the morphology from uniform fibers to bonded structures.

In solvent-cast films, CGA incorporation introduces a balance between matrix densification and surface roughness. While neat biopolymer films typically display smooth surfaces, the addition of CGA often induces microstructural heterogeneity. For instance, in agar-based films, CGA occupies interstitial spaces within the polymer network. This leads to the formation of surface wrinkles and protrusions [62]. Comparable surface coarsening has also been observed in silk fibroin/cellulose nanocrystal composites [72].

**Table 2:** Effect of CGA incorporation on mechanical and structure-related properties of films.

Matrix System	CGA Form/Concentration	Mechanical Properties	Barrier & Surface Properties	Optical/Thermal Behavior	Ref.
Starch/sweet whey	Free CGA (0.5–3% *w/w*)	Thickness: First ↑ 64% (2% CGA) and then returned to the control level (3% CGA) TS: ↓ 24% (3% CGA) EAB: First ↑ 48% (1% CGA) and then returned to the control level (3% CGA)	Water solubility: ↑ 25% (3%) Water absorption: ↑ 11% (3%) Swelling rate: First ↑ 34% (1% CGA) and then ↓ 35% (3% CGA) vs. control WCA: ↓ 20% (3% CGA)	ΔE: ↑ 88% (3%) UV: ↑ shielding to 100% (3%) Opacity: ↑ 17.5% darkening (3%)	[55]
Carrageenan/gelatin	Cu-CGA nanoparticles (0.01–0.15 mg/mL)	Thickness: ↑ 51% (0.15 mg/mL) TS: ↑ 19% (0.15 mg/mL) EAB: ↓ 53% (0.15 mg/mL)	Water solubility: ↑ 22% (0.15 mg/mL) WVP: ↓ 30% (0.15 mg/mL) WCA: ↑ 13% (0.15 mg/mL)	ΔE: ↑ 175% (0.15 mg/mL) vs. control Thermal: ↑ Maximum thermal degradation temperature	[66]
Pullulan/gelatin	Free CGA (1.0 mg mL^−1^)	Thickness: ↑ 30% TS: ↓ 20% EAB: ↑ 600%	WVP: ↑ ~2.5% WCA: Unchanged	ΔE: ↓ 45% UV: ↑ 17% shielding Thermal stability: improved	[79]
	Chitosan/rhamnolipid/CGA nanoparticle (1.0 mg mL^−1^)	Thickness: ↑ 12% TS: ↑ 13% EAB: ↑ 800%	WVP: Unchanged WCA: Unchanged	ΔE: ↑ 25% UV: ↑ 9% shielding Thermal stability: improved	
Chitosan/gelatin	Free CGA (7.35–36.75 wt %)	Thickness: ↑ 25% TS: ↓ 27% EAB: ↓ 41%	Water solubility: ↑ 43% WVP: ↑ 30%	Opacity: ↑ 210%	[61]
	CGA–graft-chitosan conjugates (1.6 g)	Thickness: Unchanged TS: ↓ 54% EAB: ↓ 70%	Water solubility: ↑ 55% WVP: ↓ 10%	Opacity: ↑ 88%	
Silk fibroin/cellulose nanocrystals	Free CGA (8.33 wt %)	Elastic modulus: ↑ 24% TS: ↑ 30% EAB: ↑ 24%	WCA: ↑ 12% WVP: ↓ 44%	Yellowness index: ↓ 56% Whiteness index: ↓ 10% Chroma: ↓ 53%	[72]
Chitosan/polycaprolactone	CGA-loaded halloysite nanotube (2–6 wt %) (nanofibers)	Diameter: ↑ 72% Elastic modulus: ↓ 52% TS: ↓ 56% EAB: ↓ 74%	WCA: ↓ 50% WVP: ↓ 40%	Thermal stability: improved	[65]
Corn starch	Free CGA (4% *w/w*)	Thickness: Unchanged TS: Unchanged EAB: Unchanged	WVP: ↑ Improved Shielding: ↑ Improved	ΔE: ↑ 95% UV: ↑ Improved	[59]
Agar	Free CGA (0.1–0.5 mg/mL)	Thickness: ↑ 32% TS: ↑ 45% EAB: ↓ 65%	WVP: Not changed WCA: ↑ 9% (0.5 mg/mL)	ΔE: ↑ 174% vs. control UV: ↑ Blocking Opacity: Unchanged	[62]
Chitosan	CaO_2_/CGA/Fe nanoparticles (0.01–0.20 mg/mL)	Thickness: ↑ 16% (0.2 mg/mL) TS: ↑ 33% EAB: ↓ 46%	WVP: ↓ 27% Oxygen permeability: ↓ 17% vs. control WCA: ↑ vs. control	ΔE: ↑ 200% Opacity: ↑ darkening UV: ↑ 100% shielding Thermal stability: Unchanged	[16]
Pure cellulose	Free CGA (450–900 ppm)	Elastic modulus: ↑ 26% SHM: ↑ 43% SAB: ↓ 20%	WCA: ↑ 15%	-	[63]
Nanocellulose	Free CGA (450–900 ppm)	Elastic modulus: ↑ 10% at 450 ppm and 3% ↓ at 450 ppm Strain hardening modulus: ↓ 19% Strain at break: ↓ 60%	WCA: ↑ 60%	-	
Polyvinylpyrrolidone	Encapsulated CGA (4% *w/w*) (nanofibers)	Elastic modulus: ↓ 90% TS: Unchanged EAB: Unchanged	WCA: ↓ 48%	Thermal: ↑ 4% denaturation temperature, melting enthalpy ↑ 12%	[77]
Polyurethane/polyvinylpyrrolidone	Encapsulated CGA (4% *w/w*) (nanofibers)	Elastic modulus: ↓ 99% TS: ↑ 1724% EAB: ↑ 2565%	WCA: ↑ 25%	Thermal: Melting enthalpy ↓ 14%	
Poly(vinyl alcohol)	CGA carbon nanodots (0.05 wt %)	TS: ↓ 7% EAB: ↓ 6%	WCA: ↑ 87% CO_2_ permeability: ↑ 9%	UV: ↑ 64% shielding	[18]
Zein	Free CGA (0.5–2.0% *w/w*)	Elastic modulus: ↑ 118% TS: ↑ 125% EAB: Unchanged	-	Thermal: ↓ 12% thermal denaturation temperature	[64]

In contrast, Li, Lin [80] reported that varying CGA concentrations did not disrupt the morphology of polyurethane (PU) films. This stability was attributed to strong hydrogen bonding between CGA and PU chains, which enhanced interfacial compatibility and enabled uniform dispersion. The presence of particulate fillers, such as Cu–CGA nanoparticles, can further modify surface morphology. These fillers may produce granular bulges due to aggregation at elevated loadings [66]. Importantly, despite increased surface roughness, cross-sectional analyses consistently reveal crack-free and cavity-free internal structures [46,49]. This indicates that CGA alters surface topology without compromising the continuity of the internal polymer network.

Morphological homogeneity is strongly influenced by processing conditions. Zhang, Zhang [59] showed that simple CGA incorporation into starch films initially caused phase separation. However, high-speed homogenization (6000–12,000 rpm) effectively eliminated these defects. This process enhances dispersion and promotes interactions between starch hydroxyl groups and CGA carboxyl groups. These findings highlight that CGA-induced heterogeneity is often a reversible processing artifact rather than an inherent material limitation. Such issues can be mitigated through optimized dispersion strategies.

#### 5.1.2. Chemical Bonding and Molecular Interactions

##### Molecular Interactions and Water Dynamics via NMR Analysis

^1^H NMR spectroscopy serves as a definitive tool for confirming the chemical grafting of CGA onto protein backbones. While physical mixing typically results in a superposition of spectra, the conjugation process induces distinct changes in the chemical environment of the protons. Fu, Wu [71] observed that the region between 5.0 and 7.5 ppm in the CGA–gelatin spectra was enlarged compared to pure gelatin. Furthermore, specific new peaks appeared in this region. The emergence of these resonances confirms that CGA was successfully conjugated rather than merely entrapped. This establishes a stable chemical linkage between the phenolic compound and the protein matrix.

Moreover, low-field NMR (LF-NMR) analysis reveals that CGA acts as a significant modulator of water mobility and matrix density in starch-based composites. The transverse relaxation time (T_2_) serves as an indicator of the molecular mobility of water within the polymer network. The addition of CGA to pure starch shifted the relaxation peaks (T_21_ and T_22_) toward shorter values. This shift reflects a restriction in proton mobility, suggesting that CGA strengthens the interactions within the starch matrix. By establishing a more compact network through hydrogen bonding, CGA effectively restricts the translational and rotational motion of water molecules. This reduces the overall water mobility within the composite system.

LF-NMR data further highlight a complex relationship between homogenization speed and water distribution (A_21_, A_22_, A_23_). Mild shear forces may initially disrupt structural integrity, weakening water-binding sites. Conversely, high mechanical energy input facilitates a reorganization of the matrix. This promotes stronger molecular interactions between starch, CGA, and water. Consequently, high-speed homogenization transforms free or semi-bound water molecules into tightly bound ones. This process yields a composite with superior water retention and a highly reinforced network structure [59].

##### Chemical Interactions and Structural Modulation via FTIR Analysis

Fourier transform infrared spectroscopy (FTIR) serves as a molecular fingerprint for elucidating interactions between CGA and biopolymer matrices. Three primary interaction mechanisms are generally identified: (i) physical hydrogen bonding in blended films, (ii) covalent conjugation in grafted matrices, and (iii) secondary structure modulation in protein-based systems. These interactions are reflected in FTIR spectra through shifts in characteristic absorption bands, the emergence of new peaks, and changes in band intensity.

In physically blended systems, CGA–matrix interactions are primarily governed by hydrogen bonding. This is commonly reflected by a decrease in peak intensity or shifts in hydroxyl and amide stretching vibrations. Chen, Zhang [81] reported a noticeable attenuation of the absorption band around 1000 cm^−1^, attributed to intermolecular hydrogen bonds between CGA and the starch matrix. This interaction reduced the availability of free hydroxyl groups along the starch chains. In polyurethane/polyvinylpyrrolidone nanofibers, Shen, Yang [77] observed a shift of the amide A band from 3420 to 3448 cm^−1^ after CGA encapsulation. This shift indicates strong interfacial hydrogen bonding between the polymer layers. Similarly, in polycaprolactone/chitosan composites, shifts in N–H and hydroxyl bands confirmed hydrogen bond formation between CGA@halloysite nanotubes and the polymer matrix [65].

In chemically grafted systems, FTIR analysis provides clear evidence of covalent integration. Fu, Wu [71] observed shifts in N–H stretching vibrations accompanied by intensified C=O and N–H bending bands. These changes confirm amide bond formation between CGA and gelatin. Similarly, Hu, Sun [61] identified characteristic amide (1642 cm^−1^) and ester (1735 cm^−1^) absorption bands in chitosan–CGA conjugates. This demonstrates dual covalent attachment through both the amino and hydroxyl groups of chitosan.

Beyond covalent bonding, CGA induces conformational rearrangements in proteins. Deconvolution of the amide I region in silk fibroin films revealed that CGA promoted a transition from random coils and α-helices to β-sheet conformations, increasing β-sheet content to 36.32% [72]. More recently, Zhang, Zeng [82] reported the formation of new chemical linkages between starch and CGA, evidenced by a distinct C=O absorption band near 1716 cm^−1^. Consistent with this observation, Li, Lin [80] identified a new C=C absorption band at 1445 cm^−1^, attributed to aromatic interactions. Finally, FTIR analysis confirms that CGA incorporation does not compromise polymer integrity, as characteristic skeletal vibrations of agar [62], polyurethane nanofibers [77], and polycaprolactone/chitosan matrices [65] remained unchanged, indicating that CGA enhances functionality while preserving the fundamental chemical framework of the films.

#### 5.1.3. Crystallinity and Molecular Arrangement

X-ray diffraction (XRD) analysis is a fundamental technique for elucidating the physical state of CGA within polymer matrices. It evaluates how CGA incorporation influences the ordered arrangement of host polymer chains. Pristine CGA exhibits a well-defined crystalline structure. This is characterized by sharp diffraction peaks arising from its periodic lattice [78]. Upon incorporation into polymers, however, the impact of CGA on crystallinity is highly matrix-dependent. This can result in the preservation, reduction, or enhancement of the crystalline structure.

In various polysaccharide and protein-based films, CGA functions as a non-disruptive filler. This is evidenced by the preservation of the native crystalline features of the matrix. For instance, in agar-based films, CGA incorporation did not generate new diffraction peaks. Instead, it caused only minor shifts in existing ones, suggesting that the crystallinity of the agar was not substantially altered [62]. Similarly, Jiang, Sheng [66] observed that incorporating Cu–CGA nanoparticles into carrageenan/gelatin films preserved the native crystal structure without producing new crystalline phases.

CGA can also promote crystallinity in a concentration-dependent manner. Hydrogen bonding between CGA phenolic hydroxyl groups and polymer chains may facilitate localized chain alignment. Such CGA-induced ordering contributes to matrix densification and has been associated with improved mechanical reinforcement in polyurethane-based films [80].

Conversely, strong intermolecular interactions—primarily hydrogen bonding and chain interpenetration—can disrupt native crystalline domains. This drives the system toward a more amorphous state. Chen, Zhang [81] demonstrated that interactions between CGA and starch chains significantly suppressed starch recrystallization. This reduction in crystallinity enhanced chain mobility and plasticization efficiency, thereby improving the functional performance of the films.

Processing conditions further modulate these outcomes. In corn starch-based films, native A-type starch (≈40% crystallinity) was largely transformed into an amorphous structure (≈2.4%) following film formation [59]. While CGA addition alone did not alter this low crystallinity, increasing the homogenization speed from 0 to 9000 rpm raised it to 13.99%. The applied mechanical shear promoted starch chain reorganization. This underscores the critical interplay between processing energy, molecular ordering, and structural performance.

#### 5.1.4. Modulation of Protein Secondary Structure

Circular dichroism (CD) spectroscopy provides a sensitive probe for monitoring conformational changes in protein matrices. CGA consistently acts as a structural perturbant. It induces secondary-structure transitions in proteins such as zein and gelatin through specific molecular interactions.

In zein-based systems, the native protein is rich in ordered α-helices. These are characterized by negative CD peaks at 208 nm (π→π*) and 221 nm (n→π*). Wang, Pan [41] demonstrated that CGA binding induces a concentration-dependent unfolding of zein. Specifically, α-helix content decreased from 61.15% in native zein to 57.59% after CGA addition. This loss of order is attributed to electrostatic interactions and hydrogen bonding. These interactions compete with the native intrachain forces that stabilize the α-helical structure.

In more complex films, the effect of CGA is non-monotonic. Chen, Liu [70] reported significant shifts in CD band intensity with varying CGA loadings in gelatin/wheat gliadin nanofibers. At an intermediate CGA level (125 mg), films retained the highest $\alpha$-helix content (14.8%). However, increasing CGA to 150 mg led to a marked collapse in structural order. In this state, α-helices dropped to 9.5%, while disordered conformations—such as random coils (27.7%)—reached their maxima. This suggests a critical threshold where excessive CGA promotes intramolecular hydrogen bonding rather than intermolecular bridging.

Overall, CD analyses confirm that CGA is an active conformational modulator. Across various systems, CGA generally reduces highly ordered α-helical structures in favor of more flexible conformations. This transition is driven by the energetic competition between CGA–protein hydrogen bonds and native stabilizing interactions.

### 5.2. Physicochemical Properties of Films

#### 5.2.1. Mechanical Behavior and Structural Integrity

**Figure 3:**
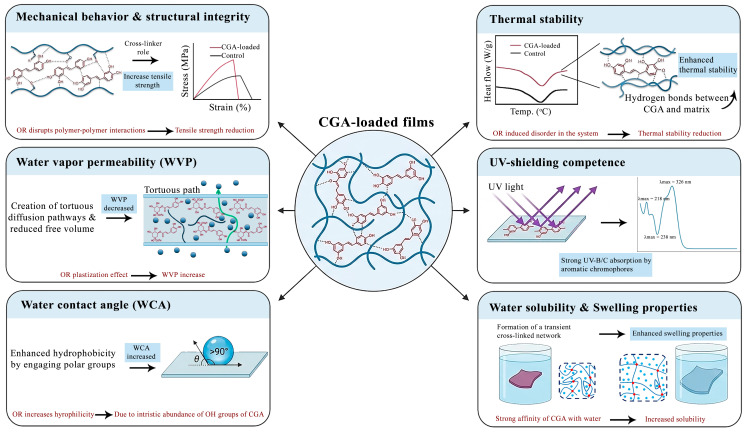
Effect of CGA on the mechanical, thermal, and barrier properties of biopolymer films.

The mechanical performance of CGA-incorporated films reflects a delicate balance between reinforcement and structural disruption. This balance is governed by the interplay between CGA concentration, matrix chemistry, and processing conditions. Rather than exerting a uniform effect, CGA displays a dual role. It may densify the polymer network through strong interfacial interactions. Conversely, at certain conditions, it may introduce discontinuities that weaken mechanical integrity (Figure 3; Table 2).

In reinforcement-dominated systems, the phenolic hydroxyl and carboxyl groups of CGA form extensive hydrogen bonding networks. In some cases, they may even form covalent linkages with polymer chains. These interactions act as effective physical cross-linking points. They enhance matrix cohesion and restrict chain mobility. Additionally, the rigid aromatic structure of CGA contributes to molecular stiffening. Collectively, these factors lead to significant improvements in tensile strength [60,62]. For instance, Wang, Pan [41] demonstrated that incorporating CGA into zein coaxial nanofibers resulted in a linear increase in both tensile strength and elastic modulus. In silk fibroin systems, Shuang, Zong [72] linked the increase in Young’s modulus to a CGA-induced conformational transition from random coils to stable β-sheet structures.

Conversely, in less compatible matrices or at excessive loadings, CGA may disrupt polymer–polymer interactions. This occurs when CGA preferentially binds to individual chains, weakening network continuity. This interference results in reduced tensile strength and elongation at break [55,61]. For example, Dai, Wang [55] reported that CGA–matrix interactions were energetically weaker than the native starch–protein bonds they replaced.

Regarding the processing conditions, Zhang, Zhang [59] provided pivotal insight into mechanical optimization. They demonstrated that processing energy can override intrinsic material limitations. While simple mixing produced negligible improvements in starch films, high-shear homogenization (9000 rpm) significantly elevated tensile strength. Similarly, Zhang, Zeng [82] showed that high-pressure homogenization (10–50 MPa) dramatically enhanced the performance of CGA–corn starch films. In their study, tensile strength increased from 1.63 to 6.29 MPa, while the elastic modulus rose from 12.02 to 160.98 MPa. This suggests that mechanical shear effectively deagglomerates particle clusters and promotes crystalline reorganization. These findings underscore that suboptimal mechanical performance often stems from insufficient dispersion rather than fundamental molecular incompatibility.

#### 5.2.2. Thermal Stability and Phase Transitions

Thermal characterization via differential scanning calorimetry (DSC) and thermogravimetric analysis (TGA) reveals that CGA influences thermal behavior in a matrix-dependent manner. Rather than serving as a universal stabilizer, CGA regulates phase transitions via two contrasting pathways. It can act as a molecular bridge in compatible systems or as a structural disruptor in sensitive protein matrices (Figure 3; Table 2).

In most biopolymer films, CGA incorporation enhances thermal stability by reinforcing interfacial interactions. The abundant functional groups of CGA promote extensive hydrogen-bond networks, increasing matrix cohesion. As a result, thermally induced molecular motion is suppressed. This delays the onset of thermal decomposition and improves resistance to heat-induced degradation [80].

Beyond stability, CGA also governs phase transitions. Its effect is dictated by the balance between chain confinement (cross-linking) and free-volume generation (plasticization). Chen, Liu [70] reported a non-monotonic glass transition temperature (Tg) response in gelatin/wheat gliadin electrospun fibers. At moderate CGA concentrations, Tg increased, reflecting the rigidification of the amorphous phase through dense hydrogen bonding. However, at higher CGA loadings, this trend reversed. Excess unbound CGA acted as a plasticizing agent, increasing segmental mobility and free volume.

A similar concentration-dependent behavior has been observed for crystallization. In polycaprolactone/chitosan systems, Zou, Zhang [65] demonstrated that CGA-loaded halloysite nanotubes served as effective heterogeneous nucleation sites at low filler contents (≤2 wt%). This led to increases in melting temperature and enthalpy of fusion. In contrast, higher loadings induced steric hindrance and agglomeration. This disrupted orderly chain folding and ultimately suppressed crystallization, reducing thermal stability.

Notably, these reinforcement mechanisms do not universally apply to all matrices. In zein coaxial nanofibers, Wang, Pan [41] reported a significant reduction in thermal denaturation temperature following CGA incorporation. Circular dichroism analysis attributed this destabilization to CGA-induced disruption of compact α-helical domains. This disruption lowered the energetic barrier for protein unfolding, leading to earlier thermal degradation.

#### 5.2.3. Optical Properties and UV-Shielding Competence

The integration of CGA into biopolymer films establishes a critical trade-off between optical transparency and photoprotection. CGA functions as a highly selective spectral filter. It effectively attenuates high-energy ultraviolet (UV) radiation while maintaining sufficient transparency in the visible spectrum. This dual functionality is governed by CGA concentration, its dispersion state, and the processing history of the film (Figure 3; Table 2).

The potent UV-blocking capability of CGA is intrinsic to its molecular architecture. The presence of catechol moieties and conjugated double bonds facilitates π→π* electronic transitions. These transitions enable efficient absorption of UV radiation in the UV-B and UV-C regions (200–315 nm). This effect is strongly dose-dependent. For instance, increasing the CGA content to 3% in sweet whey/starch films resulted in complete UV extinction at 280 and 315 nm [55]. This provide effective protection against photo-induced lipid oxidation without compromising visual transparency.

Most CGA-functionalized films retain high transparency in the visible region (400–700 nm). Starch and agar-based films, for example, maintain transmittance values between 58% and 70% at 600 nm, ensuring the product remains clearly visible [55,62]. However, CGA inevitably alters the colorimetric profile. A consistent decrease in lightness (L*) and an increase in yellowness (b*) and redness (a*) are typically observed. This is due to the intrinsic yellow–brown hue of oxidized polyphenols. While these changes yield a perceptible total color difference (ΔE > 1), this “amber tint” can be functionally advantageous. It acts as a secondary barrier for light-sensitive components like pigments and vitamins.

Optical performance is also governed by processing conditions. Increasing mechanical shear during film preparation enhances UV-shielding efficiency. This is primarily due to shear-induced particle size reduction, which increases the effective surface area for light absorption [59]. Additionally, while physical blending often increases opacity due to micro-aggregation, chemical grafting markedly improves transparency [61]. Covalent attachment suppresses aggregate formation, yielding films that remain optically clear while retaining functional activity.

#### 5.2.4. Water Vapor Barrier Performance

The modulation of water vapor permeability (WVP) in CGA-functionalized films is dictated by a competitive equilibrium. This balance exists between the “tortuosity effect,” which obstructs diffusion, and the “plasticization effect,” where excess free volume facilitates moisture transport. The final barrier performance depends on the incorporation strategy and the applied processing energy (Figure 3; Table 2).

In nanocomposite architectures, CGA consistently functions as a barrier enhancer. The integration of Cu–CGA nanoparticles or CGA-loaded halloysite nanotubes significantly reduces WVP by establishing a tortuous diffusion pathway. These impermeable nanofillers occupy matrix voids and reduce porosity. This forces water molecules to traverse a meandering path, thereby extending the diffusion lag time [65,66]. Similarly, in silk fibroin systems, Shuang, Zong [72] reported that the addition of CGA and cellulose nanocrystals reduced WVP. This was attributed to a dense hydrogen-bonding network that “locks” available hydrophilic sites.

The mode of incorporation—chemical versus physical—plays a decisive role. Covalent grafting of CGA onto chitosan backbones reduces WVP by consuming hydrophilic amino and hydroxyl groups [61]. In contrast, simple physical blending can have a detrimental effect. Unreacted CGA may behave as a hydrophilic impurity, generating excess free volume that functions as moisture diffusion channels. Crucially, Zhang, Zhang [59] highlighted that processing energy is a critical variable. While simple incorporation yielded negligible improvements, high-shear homogenization (9000 rpm) markedly reduced WVP. This improvement stems from the refinement of starch granules and the promotion of crystalline domains. However, the relationship between shear and barrier performance is parabolic: excessive shear (12,000 rpm) degraded the polymer network and reduced crystallinity, leading to a renewed increase in WVP.

Consequently, mitigating the drawbacks associated with the hydrophilicity of CGA requires a strategic approach. Optimal barrier performance is achieved not through simple addition, but by: (1) utilizing nanocarriers to increase diffusion path tortuosity, (2) employing shear-induced crystallization to densify the polymer matrix, or (3) optimizing grafting density to reduce moisture uptake without compromising the structural integrity of the polymer network.

#### 5.2.5. Surface Wettability and Hydrophobicity

Water contact angle (WCA) serves as a key indicator of surface energy and the film’s interaction with moisture. The incorporation of CGA exerts a variable effect on surface wettability, governed by the interplay between matrix chemistry, filler morphology (free CGA vs. nanoparticles), and surface distribution. Specifically, CGA may enhance hydrophobicity by engaging polar groups within a dense network, or increase hydrophilicity due to its inherent abundance of hydroxyl groups (Figure 3; Table 2).

In matrices where CGA acts primarily as a cross-linker, a distinct increase in WCA is observed, indicating improved water resistance. This effect is driven by the consumption of free hydrophilic groups (–OH, –NH_2_) on the polymer chains through extensive hydrogen bonding with CGA. Previous studies have shown that incorporating Cu–CGA nanoparticles into carrageenan/gelatin films, as well as CGA into agar-based films, increased the WCA [62,66]. In these systems, the competition between CGA and water molecules for binding sites effectively lowers the surface affinity for moisture. A structural rationale for this was provided by Shuang, Zong [72] in silk fibroin systems, where the addition of CGA (alongside cellulose nanocrystals) elevated the WCA to 90.1°. This shift was attributed to the CGA-induced transition towards β-sheet formations, a stable crystalline conformation that inherently enhances hydrophobic barriers.

Conversely, in highly hydrophilic or loosely structured matrices, CGA can promote surface wetting. Due to its polar nature, excess CGA segregating at the film surface acts as a wetting agent. In electrospun PVP films, CGA encapsulation dramatically reduced the initial WCA from 105.0° to 55.2°, followed by a rapid decline to 29.2° within 3 s [77]. This behavior reflects the rapid dissolution of surface-exposed CGA, facilitating water spreading. Similarly, Dai, Wang [55] reported that adding 0.5% CGA to sweet whey/starch films caused a sharp decrease in WCA from 104.1° to 69.7°, attributed to the disruption of native hydrophobic interactions within the protein network. However, notably, the WCA partially recovered at higher loadings (up to 83.5°), suggesting that once a critical concentration is reached, the formation of a stable cross-linked network can override the initial hydrophilic effect.

Furthermore, wettability often follows a non-linear, concentration-dependent trajectory. Chen, Liu [70] observed in gelatin/gliadin films that moderate CGA doses (25–75 mg) increased WCA by consuming hydrophilic sites, whereas excessive doses caused a decline, signaling a “saturation threshold” where free hydrophilic CGA accumulates on the surface. Ultimately, surface topography also modulates this property; Zou, Zhang [65] attributed increased WCA in polycaprolactone/chitosan mats to the enhanced surface roughness provided by halloysite nanotubes. Consequently, the modulation of WCA dictates application suitability: hydrophobic films are ideal for moisture-sensitive products [62], while hydrophilic surfaces may offer advantages in specific anti-fouling applications by creating an unfavorable hydration layer for bacterial adhesion.

#### 5.2.6. Water Solubility and Swelling Behavior

The interaction between CGA and water within biopolymer matrices is governed by two competing factors: the intrinsic hydrophilicity of CGA and its ability to form physical or chemical networks that restrict water accessibility. Accordingly, the effect of CGA on water resistance is highly formulation-dependent, differing markedly among blended, grafted, and nanocomposite systems (Figure 3; Table 2).

In physical blends such as sweet whey/starch films, CGA often behaves as a hydrophilic additive that compromises water resistance. Dai, Wang [55] reported that CGA addition linearly increased water solubility and absorption. Although CGA forms new hydrogen bonds with the matrix, its strong affinity for water and disruption of polymer–polymer interactions result in higher solubility overall. Notably, swelling followed a non-linear trend, peaking at 1% CGA loading (from 108.1% to 222.3%), suggesting the formation of a transient cross-linked network capable of retaining large amounts of water. While reduced water resistance is undesirable for liquid packaging, the high swelling capacity makes such films suitable for internal coatings that regulate humidity and suppress microbial growth in dry or semi-dry foods. An enhanced swelling rate can effectively reduce moisture accumulation within the packaging environment.

Hu, Sun [61] compared physically incorporated and chemically conjugated chitosan films. Both approaches significantly reduced moisture content relative to pure chitosan. In incorporated films, CGA occupied hydrophilic hydroxyl and amino sites, limiting water interaction. In grafted films, covalent bonding disrupted the original hydrogen-bonding network of chitosan, further reducing moisture retention. Despite this, both systems exhibited increased water solubility. In grafted films, disruption of strong intermolecular interactions facilitated chain dissolution, whereas in incorporated films, rapid dissolution of free CGA accelerated matrix disintegration.

By contrast, CGA-incorporated nanocomposites provide an effective route to enhanced water resistance. Jiang, Sheng [66] showed that incorporating Cu-CGA nanoparticles into carrageenan/gelatin matrices reduced water solubility from 27.01% to 20.89%. This improvement was attributed to the lower solubility of Cu-CGA nanoparticles relative to free CGA and the formation of a dense, cross-linked network with the polymer matrix, as confirmed by FTIR analysis. Such networks act as physical barriers to water penetration while consuming free hydrophilic groups, effectively restricting water mobility.

#### 5.2.7. Structure–Function Analysis of Physicochemical Properties of the Packaging Films

A structure–function-oriented comparison of CGA-incorporated films reveals that their mechanical, barrier, surface, optical, and thermal properties are governed not only by CGA concentration, but more critically by its incorporation mode (free, encapsulated, grafted, or nanoparticle-assisted) and the structural organization of the polymer matrix. Distinct performance patterns emerge across systems, highlighting trade-offs between mechanical reinforcement, flexibility, barrier efficiency, and optical functionality.

From a mechanical standpoint, the effect of CGA is highly system-dependent. In polysaccharide-based matrices such as starch/sweet whey and pullulan/gelatin, incorporation of free CGA generally leads to plasticization effects, reflected by reduced tensile strength (TS ↓ 20–24%) and significantly increased elongation at break (EAB ↑ up to 600%) [55,79]. In contrast, nanostructured or nanoparticle-assisted systems can induce reinforcement. For example, carrageenan/gelatin films containing Cu–CGA nanoparticles exhibited TS enhancement of 19%, albeit with reduced flexibility (EAB ↓ 53%) [66], indicating a transition toward a more rigid network due to nanoparticle–polymer interactions. Similarly, silk fibroin/cellulose nanocrystals films showed simultaneous improvements in modulus (+24%), TS (+30%), and EAB (+24%) [72], representing one of the few systems achieving balanced mechanical reinforcement. Moreover, the observed changes in mechanical parameters are also intrinsically linked to the significant increases in film thickness (up to 51% in nanoparticle-based systems), which reflects the incorporation of bulky fillers and the expansion of the polymer network.

A key structural insight emerges when comparing free vs. grafted CGA systems. In chitosan/gelatin films, free CGA reduced TS (−27%) and EAB (−41%), whereas CGA–grafted chitosan caused even more pronounced reductions (TS ↓ 54%, EAB ↓ 70%) [61]. Despite improved barrier properties (WVP ↓ 10%), grafting restricts polymer chain mobility, leading to embrittlement. This highlights a critical trade-off: covalent integration enhances structural cohesion and barrier performance but compromises flexibility.

Electrospun nanofiber systems exhibit another distinct behavior. In chitosan/polycaprolactone nanofibers containing CGA-loaded halloysite nanotubes, mechanical properties deteriorated significantly (TS ↓ 56%, EAB ↓ 74%, modulus ↓ 52%) [65], due to disrupted fiber continuity and stress concentration points. Conversely, in polyurethane/polyvinylpyrrolidone sandwich nanofibers, extreme improvements in TS (+1724%) and EAB (+2565%) were observed [77], demonstrating that hierarchical fiber architectures can dramatically enhance mechanical performance when load transfer between layers is optimized.

Barrier and surface properties show more consistent trends linked to CGA’s polarity and interaction with the matrix. In many hydrophilic systems (e.g., starch, chitosan), CGA increases water affinity, leading to higher water solubility (+25–55%) and reduced water contact angle (WCA ↓ up to 50%) [55,61,65]. However, in nanoparticle-based systems, barrier properties are often improved. For instance, carrageenan/gelatin films with Cu–CGA nanoparticles showed WVP reduction of 30% [66], while silk fibroin/cellulose nanocrystals films achieved WVP reduction of 44% [72], indicating enhanced tortuosity and reduced diffusion pathways. Similarly, CaO_2_/CGA/Fe nanoparticle chitosan films reduced both WVP (−27%) and oxygen permeability (−17%) [16], demonstrating multifunctional barrier enhancement.

Encapsulation strategies also significantly influence barrier performance. In CGA-loaded halloysite nanotube systems, WVP decreased by 40% despite reduced hydrophobicity (WCA ↓ 50%) [65], suggesting that nanofiller-induced tortuosity dominates over surface wettability effects. In contrast, simple free CGA incorporation in pullulan/gelatin films slightly increased WVP (~2.5%) [79], indicating that without structural reinforcement, CGA may disrupt matrix packing.

Optical and UV-barrier properties are consistently enhanced by CGA incorporation due to its phenolic chromophores. Many systems achieved substantial increases in color difference (ΔE up to 200%) and opacity (up to +210%) [16,61], reflecting strong light absorption and film darkening. Notably, complete or near-complete UV shielding (≈100%) was achieved in starch-based and nanoparticle-containing films [16,55], making them highly suitable for light-sensitive food applications. However, this often comes at the cost of transparency, which may limit consumer acceptance depending on application.

Interestingly, some nanostructured systems improve optical quality differently. For example, silk fibroin/cellulose nanocrystals films showed reductions in yellowness (−56%) and chroma (−53%) [72], suggesting more uniform CGA dispersion and reduced aggregation. This highlights that optical behavior is not solely dictated by CGA presence but by its dispersion state within the matrix.

Thermal properties further reflect structure–function relationships. In most systems, CGA improves thermal stability through hydrogen bonding and radical scavenging. For instance, carrageenan/gelatin films with Cu–CGA nanoparticles exhibited increased degradation temperature [66], while pullulan/gelatin and nanoparticle-containing systems also showed improved thermal stability [79]. However, in protein-based systems such as zein, CGA reduced denaturation temperature by 12% [64], indicating that CGA may disrupt protein folding depending on interaction strength.

Overall, quantitative benchmarking reveals that no single system optimizes all properties simultaneously. Nanocomposite and nanoparticle-assisted systems provide the best barrier performance (WVP reduction up to 44%) and improved mechanical strength, while electrospun hierarchical structures can achieve extreme mechanical enhancement when properly designed. Free CGA systems favor flexibility but often compromise strength and barrier properties, whereas grafted systems improve barrier performance at the expense of mechanical ductility. Optical and UV-shielding performance are consistently enhanced across nearly all systems, with UV blocking approaching 100% in optimized formulations.

In summary, performance follows a structure-dependent hierarchy: nanoparticle-assisted ≈ nanocomposite > grafted > encapsulated > free CGA systems for barrier and strength properties, while the reverse trend is often observed for flexibility. These findings underscore that rational design of CGA incorporation strategy and matrix architecture is essential to tailor multifunctional packaging films with balanced mechanical integrity, barrier efficiency, and optical functionality.

### 5.3. Bioactive Functional Attributes

#### 5.3.1. Antioxidant Activity and Radical Scavenging Mechanism

The antioxidant activity of CGA originates from its molecular structure. It contains multiple phenolic hydroxyl groups capable of directly scavenging reactive oxygen species (ROS) through hydrogen atom donation. This process effectively terminates oxidative chain reactions and stabilizes radical intermediates. Additionally, the steric hindrance of the CGA molecule limits self-oxidation. This reduces the likelihood of pro-oxidant behavior and minimizes secondary oxidative damage to food components. The combined presence of phenolic hydroxyl groups and conjugated double bonds further enhances electron-donating ability. This enables efficient electronic interactions with the d-orbitals of metal ions, contributing to metal-chelating antioxidant effects [16].

Consistent with these characteristics, incorporating CGA converts biopolymer matrices into active antioxidant films. CGA-loaded films have repeatedly demonstrated strong radical-scavenging capacity across various processing conditions. They provide sustained inhibition of lipid and protein oxidation in packaged foods [55,58]. Beyond direct radical neutralization, CGA also protects food macromolecules from oxidative degradation. In protein-rich systems, such as fish, CGA intercepts reactive species that would otherwise oxidize sulfhydryl groups (–SH) into disulfide bonds (–S–S–). This prevents protein aggregation and quality deterioration [58]. The overall impact of CGA incorporation on the functional properties of packaging films is summarized in Table 3.

**Table 3:** Functional bioactivity of CGA-incorporated films: Antioxidant capacity and antimicrobial efficacy across all studied matrices.

Matrix System	Antioxidant Activity	Antimicrobial Efficacy	Key Findings	Ref.
CGA–starch/sweet whey film	DPPH: Increased dose-dependently to 76.4% (3% CGA). ABTS: Increased dose-dependently to 92.7% (3% CGA)	Antibacterial rate against *E. coli*: Dose-dependent efficacy, reaching 92.7% at 3% CGA.	Antimicrobial activity: Breaking down the cell’s barrier, halting its reproduction, and disrupting its essential internal processes. Antioxidant activity: Directly neutralizing free radicals with its chemical structure, and boosting the body’s internal antioxidant defense systems.	[55]
CGA–corn starch film	DPPH• scavenging activity at 4% CGA: 90.96%	Antibacterial rate at 4% CGA: *E. coli*: 89.91–93.48% *S. aureus*: 97.31–98.91%	Antimicrobial activity: Demonstrated greater efficacy against *S. aureus* than against *E. coli*, likely attributable to differences in cell wall composition. Antioxidant activity: CGA donating hydrogen atoms (H•) to reduce the radical DPPH• to non-radical DPPH-H. CGA’s activity retained after physical treatment.	[59]
Cu-CGA-nanoparticles carrageenan/gelatin film	DPPH• scavenging activity at 0.15 mg/mL Cu-CGA: 65.68%	Antibacterial rate at 0.15 mg/mL Cu-CGA: *E. coli*: 91.64% *S. aureus*: 80.23% Photothermal killing (NIR irradiation): *S. aureus*: near-complete inhibition *E. coli*: near-complete inhibition	Antimicrobial activity: Results from the combined effect of copper nanoparticles’ antimicrobial properties and NIR-induced rapid photothermal damage (cell shrinkage and structural degradation). Antioxidant activity: Phenolic -OH groups in Cu-CGA NPs donate hydrogen atoms (H•) to reduce/inhibit free radicals.	[66]
CaO_2_/CGA/Fe nanoparticles chitosan film	-	Antibacterial/antifungal rate at 0.2 mg/mL nanoparticles: *E. coli*: 60% *S. aureus*: 57.1% *Penicillium italicum*: 58.3% Photothermal killing (NIR irradiation): *S. aureus*: complete inhibition *E. coli*: complete inhibition *Penicillium italicum*: complete inhibition	Antimicrobial activity: high activity after NIR irradiation was due to the combined action of chitosan’s inherent antibacterial properties and photothermally enhanced nanozyme catalysis. Photothermal regulation of POD-like activity further increases ROS production, boosting antifungal efficacy.	[16]
CGA–zein nanofiber film	DPPH: Increased dose-dependently to 72% (2% CGA). ABTS: Increased dose-dependently to 66% (2% CGA)	Bacterial inhibition: Small inhibition zone at 2.0% CGA	Antimicrobial activity: Activity depended on CGA content and its diffusion distance to the fiber surface. Antioxidant activity: Optimizing CGA content enhances the antioxidant capacity of coaxial nanofiber films for active packaging.	[64]
Chitosan/rhamnolipid/CGA nanoparticle pullulan/gelatin film	DPPH: 91% ABTS: 97.29% (1.0 mg mL^−1^)	Antibacterial rate at 1.0 mg mL^−1^: *E. coli:* 92.14% *S. aureus*: 98.72%	Antimicrobial activity: CGA disrupts cell membrane integrity to inhibit bacterial growth. Antioxidant activity: CGA-incorporated films exhibit outstanding antioxidant activity, suitable for food packaging.	[79]
CGA-cellulose (pure/nano) film	Trolox equivalent antioxidant capacity (TEAC) per 1 cm^2^ film surface: CGA-cellulose film at 900 ppm: 410 mg/L CGA-nanocellulose film at 900 ppm: 595 mg/L	Antibacterial/antifungal efficacy: High growth inhibition of *B. cinerea, E. coli*, and *S. aureus*. No evident inhibition of *Penicillium* sp.	Antimicrobial activity: Evident at higher concentrations in cellulose film, but effective at lower concentrations in nanocellulose film. No inhibition of *Penicillium* sp. suggests composite biodegradability via fungal cellulolytic/phenolic-deactivating activity. Antioxidant activity: After 7-day mixing, activity increased up to 5-fold (CGA-nano), attributed to strong CGA binding and prolonged release.	[63]
CGA–chitosan/gelatin films (CGA–grafted-chitosan conjugates and CGA-incorporated-chitosan)	DPPH CGA–grafted-chitosan: 87% CGA-incorporated-chitosan: 77%	Inhibition zone CGA–grafted-chitosan: *E. coli:* 11.20 mm *S. aureus*: 17.61 mm CGA-incorporated-chitosan: *E. coli:* 10.96 mm *S. aureus*: 14.47 mm	Antibacterial activity: it was higher in CGA–grafted-chitosan films, correlating with a higher total release rate. Antioxidant activity: The enhanced activity is attributed to CGA moieties in the chitosan backbone. The DPPH scavenging of CGA–grafted-chitosan films was significantly higher due to higher release rate.	[61]
CGA-assisted dopamine–sodium alginate composite nanofiber	DPPH: 84% at 16 mg mL^−1^ CGA	Antibacterial rate at 8 mg mL^−1^: *E. coli:* 93.08% *S. aureus*: 88.83%	Antibacterial activity: it was due to (1) porous nanofiber surface increasing bacterial adhesion sites and (2) continuous CGA release disrupting cell membranes/walls and impairing integrity. Antioxidant activity: CGA acts as a free radical scavenger, with its antioxidant activity primarily attributed to its abundant phenolic hydroxyl groups.	[83]
CGA–gelatin/wheat gliadin electrospun film	DPPH: 92.1% ABTS: 98.2% (150 mg)	Antibacterial rate at 150 mg: *E. coli:* 16.1% *S. aureus*: 17.5% *S. putrefaciens:* 56.2%	Antibacterial activity: CGA as a phenolic compound that disrupts cell membrane structure and function, increasing permeability and leading to cell death. Antioxidant activity: Linked to CGA’s phenolic –OH groups. The retained activity confirms successful encapsulation and stability of CGA through acid/alcohol dissolution and high-voltage electrospinning.	[70]
CGA–gelatinized starch-based emulsion	DPPH: 85.28% ABTS: 96.61% (0.1 mg mL^−1^)	Antibacterial rate at 0.1 mg mL^−1^: *S. putrefaciens:* 99.4%	Antibacterial activity: Observed cell membrane deformation, shrinkage, and rupture indicate direct physical/chemical damage induced by CGA-loaded oil interfacing with the bacterial envelope. Antioxidant activity: Phenolic -OH groups in CGA acting as hydrogen atoms (H•) donors to neutralize free radicals via hydrogen atom transfer (HAT).	[84]
CGA–silk fibroin/cellulose nanocrystals film	-	Antibacterial/antifungal rate at 8.33 wt %: *E. coli:* 99.66% *S. aureus*: 91.07% *R. stolonifera*: complete inhibition	Antibacterial activity: CGA kills bacteria by mediating irreversible membrane permeability changes, leading to loss of membrane potential and leakage of cytoplasmic macromolecules.	[72]
CGA-loaded halloysite nanotube (CGA@HNTs) chitosan/polycaprolactone nanofiber film	DPPH: 51.40% (6% CGA@HNTs)	Inhibition zone at 6% CGA@HNTs: *E. coli:* 21.67 mm *S. aureus*: 19.71 mm Bactericidal kinetics after 6 h at 6% CGA@HNTs: *E. coli:* 50.7% *S. aureus*: 39.4%	Antibacterial activity: Increased with higher CGA@HNTs ratio in the fibrous mats. Gram-negative bacteria were more susceptible than Gram-positive due to differences in membrane arrangement. Antioxidant activity: Time-dependent increase in DPPH scavenging due to sustained CGA release from fibers.	[65]
CGA–polyvinylpyrrolidone nanofiber film (CGA-loaded film)	DPPH: 49.4% (4% *w/w*)	Inhibition zone at 4% CGA: *E. coli:* 14.42 mm *S. aureus*: 13.17 mm *B. cereus*: 13.40 mm Bactericidal kinetics *E. coli:* 12.39% *S. aureus*: 32.79% *B. cereus*: 16.06%	Antibacterial activity: CGA-loaded film > sandwich film in antibacterial inhibition due to greater CGA release. Antioxidant activity: CGA-loaded film > sandwich-structured film → polyurethane outer layers protect CGA in polyvinylpyrrolidone core, enabling sustained release.	[77]
CGA–polyurethane/polyvinylpyrrolidone nanofiber film (Sandwich-structured film)	DPPH: 28.5% (4% *w/w*)	Inhibition zone at 4% CGA: *E. coli:* 11.38 mm *S. aureus*: 11.06 mm *B. cereus*: 11.04 mm Bactericidal kinetics *E. coli:* 11.24% *S. aureus*: 20.10% *B. cereus*: 13.95%		
CGA–polyvinyl alcohol-based nanofiber film	DPPH: 57.6% (5% *w/w*)	Microbiocidal effect at 5% *w/w* CGA after 48 h: *E. coli:* ~2 log CFU/mL *S. aureus*: ~2.5 log CFU/mL	Antibacterial activity: Primary mechanism: K^+^ efflux → membrane permeabilization → nucleotide leakage. Secondary effects: Outer membrane binding, cation chelation, homeostasis disruption, and nutrient exchange blockage. Antioxidant activity: The antioxidant activity of CGA is based on its ability to donate hydrogen atoms (H•) to the radical DPPH•, converting it into a semiquinone radical or quinone form.	[78]

In physically blended systems, antioxidant performance typically follows a concentration-dependent trend. For example, the DPPH scavenging activity of zein-based films increased from 16% to 72% at 2.0% CGA incorporation [41]. However, this enhancement often reaches a plateau beyond a critical concentration. This ceiling effect is generally attributed to the saturation of accessible phenolic hydroxyl groups at the film surface [70]. These findings emphasize that sustained efficacy depends more on controlled CGA availability than on maximal loading.

Film architecture also governs antioxidant kinetics by modulating CGA release. Sandwich-structured films initially exhibited lower scavenging activity than monolayer films. However, they provided prolonged protection through controlled release, reaching higher scavenging levels over time [77]. A similar response was observed in polycaprolactone/chitosan mats incorporating CGA-loaded halloysite nanotubes. In these systems, the nanotubes functioned as reservoirs for sustained CGA release [65].

Finally, methodological factors can influence measured performance. Dai, Wang [55] reported higher ABTS scavenging (95.7%) than DPPH scavenging (76.4%) in starch films. This discrepancy was attributed to differential swelling behavior in the aqueous versus methanolic media.

#### 5.3.2. Antimicrobial Efficacy

CGA exhibits broad-spectrum antimicrobial activity against bacteria, molds, and yeasts. A dominant antibacterial mechanism involves the disruption of the microbial cell membrane. This leads to irreversible increases in permeability and cellular damage. In Gram-negative bacteria, membrane stability relies on divalent cations that bridge lipopolysaccharides and proteins. As an anionic polyphenol, CGA can chelate these cations. This weakens membrane integrity and compromises the barrier function [51].

In parallel, CGA compromises fungal membrane integrity by targeting ergosterol biosynthesis. Specifically, CGA suppresses the expression and activity of key sterol biosynthetic enzymes, such as *ERG4* and *ERG6*. This lead to reduced ergosterol content and increased membrane permeability [55]. Mitochondrial dysfunction emerges as a convergent antifungal mechanism. In yeasts, CGA induces membrane depolarization and excessive ROS production, eventually triggering apoptosis-like cell death [56].

Incorporating CGA into film matrices imparts broad-spectrum antimicrobial functionality. Across microbial groups, membrane destabilization remains the central mechanism. CGA induces irreversible permeability changes that cause the leakage of electrolytes and cytoplasmic macromolecules. Ungolo, Ruggeri [78] further proposed that CGA binds to bacterial outer membranes via electrostatic interactions and chelates cations essential for membrane homeostasis. In addition to membrane damage, CGA interferes with intracellular metabolic pathways and cell cycle progression, ultimately leading to metabolic dysfunction and growth inhibition [55]. These effects have been directly visualized by SEM, revealing severe membrane collapse and pronounced morphological deformation in treated *S. aureus* and *E. coli* cells [72].

Antimicrobial efficacy is also influenced by the incorporation mode. Chemically conjugated CGA films often exhibit higher antibacterial activity than blended counterparts due to controlled release [61]. Fu, Wu [71] confirmed that the conjugation process does not compromise CGA’s intrinsic function. However, excessive structural barriers, such as in sandwich-structured films, can limit effectiveness by preventing CGA from reaching bactericidal concentrations [77].

Beyond antibacterial effects, CGA-loaded films demonstrate strong antifungal activity. For instance, silk fibroin films containing CGA completely suppressed the spore germination of *Rhizopus stolonifer* [72]. This antifungal behavior parallels antibacterial mechanisms, where CGA-induced membrane permeabilization triggers spore lysis.

#### 5.3.3. Structure–Function Analysis of Antioxidant and Antimicrobial Performance

A structure–function-based analysis of CGA-incorporated films clearly demonstrates that their functional performance is not solely governed by CGA concentration, but is strongly dependent on its incorporation strategy (free, encapsulated, grafted, or nanostructured) and the physicochemical characteristics of the polymeric matrix. Systems that simultaneously enable CGA stabilization, controlled release, and high interfacial area generally exhibit superior antioxidant and antimicrobial performance. However, highly protective architectures (e.g., sandwich structures) may reduce immediate bioactivity due to restricted CGA diffusion.

From an antioxidant perspective, the highest radical scavenging capacities are predominantly observed in electrospun and nanostructured systems. For instance, CGA–gelatin/wheat gliadin electrospun films exhibited DPPH and ABTS scavenging activities of 92.1% and 98.2%, respectively [70], representing one of the highest antioxidant performances reported. Similarly, chitosan/rhamnolipid/CGA nanoparticle pullulan/gelatin films achieved DPPH and ABTS values of 91% and 97.29% [79], while CGA–corn starch films reached 90.96% DPPH scavenging at 4% CGA [59]. These results highlight the critical role of nanostructuring and polymer compatibility in enhancing CGA accessibility and radical quenching efficiency.

In terms of antimicrobial efficacy, the most effective systems are those combining efficient CGA release with intrinsic or synergistic antimicrobial components. For example, CGA-silk fibroin/cellulose nanocrystals films exhibited antibacterial rates of 99.66% against *E. coli* and 91.07% against *S. aureus*, along with complete inhibition of *R. stolonifera* [72], representing one of the strongest antimicrobial profiles. Likewise, chitosan/rhamnolipid/CGA nanoparticle pullulan/gelatin films showed high inhibition rates (92.14% for *E. coli* and 98.72% for *S. aureus*) [79], while gelatinized starch-based emulsions achieved 99.4% inhibition against *S. putrefaciens* [84]. These findings indicate that matrix composition and synergistic interactions significantly amplify antimicrobial outcomes.

A key structural determinant is the mode of CGA incorporation. Grafting strategies consistently outperform physical incorporation. In CGA–chitosan/gelatin films, grafted systems exhibited higher DPPH scavenging (87%) compared to incorporated systems (77%), along with larger inhibition zones against *E. coli* (11.20 vs. 10.96 mm) and *S. aureus* (17.61 vs. 14.47 mm) [61]. This enhancement is attributed to increased CGA availability and controlled release from the polymer backbone, demonstrating the superiority of covalent integration in optimizing functional performance.

Encapsulation-based systems provide a balance between CGA protection and sustained release. For instance, CGA-loaded halloysite nanotube (CGA@HNTs) chitosan/polycaprolactone nanofiber films showed moderate antioxidant activity (DPPH: 51.40%) but strong antimicrobial performance, with inhibition zones of 21.67 mm against *E. coli* and 19.71 mm against *S. aureus* [65]. This suggests that antioxidant and antimicrobial properties do not necessarily correlate directly, and can be selectively modulated through structural design.

Comparing fabrication techniques, electrospun nanofiber systems generally outperform conventional cast films due to their high surface area, porous morphology, and enhanced CGA diffusion. For example, CGA–polyvinylpyrrolidone nanofiber films exhibited higher antioxidant (49.4% DPPH) and antimicrobial activity (14.42 mm inhibition zone for *E. coli*) than sandwich-structured polyurethane/polyvinylpyrrolidone films (28.5% DPPH; 11.38 mm) [77]. The reduced performance of sandwich structures reflects diffusion limitations imposed by protective outer layers.

The nature of the biopolymer matrix further modulates functionality. Chitosan-based systems benefit from intrinsic antimicrobial activity, resulting in synergistic effects when combined with CGA. In contrast, cellulose/nanocellulose matrices primarily enhance CGA binding and long-term stability; for example, TEAC values reached 595 mg/L per cm^2^ in CGA-nanocellulose films compared to 410 mg/L in conventional cellulose films [63], indicating improved antioxidant retention and sustained release.

Protein-based matrices such as zein and wheat gliadin offer advantages in electrospinning and nanofiber formation. CGA–zein nanofiber films achieved DPPH and ABTS values of 72% and 66%, respectively [64], with performance strongly influenced by CGA diffusion distance within the fiber network. Meanwhile, CGA–gelatin/wheat gliadin electrospun films exhibited excellent antioxidant capacity but comparatively lower antibacterial activity against certain strains, suggesting that these systems are optimized more for oxidative stabilization than microbial inhibition.

Hybrid systems incorporating photothermal or nanozyme components (e.g., Cu-CGA or CaO_2_/CGA/Fe nanoparticles) demonstrate exceptional antimicrobial activity under external stimulation. For example, Cu-CGA films achieved near-complete bacterial inhibition under NIR irradiation, despite moderate baseline antioxidant activity (65.68% DPPH) [66]. Similarly, CaO_2_/CGA/Fe nanoparticle systems showed complete inhibition of bacteria and fungi under photothermal conditions [16]. However, these systems should be interpreted as multifunctional active packaging platforms rather than purely CGA-driven systems.

Overall, quantitative benchmarking indicates that the highest antioxidant performance (>90% DPPH/ABTS) is achieved in electrospun protein/polysaccharide hybrid systems, while the strongest antimicrobial effects (>99% inhibition) are observed in nanocomposite and synergistic matrices. Structurally, performance follows the general trend: grafted > encapsulated > physically incorporated > diffusion-limited sandwich systems in terms of immediate bioactivity, whereas nanocellulose and protected systems offer advantages in long-term stability. These findings highlight that rational design of CGA delivery architecture is essential to tailor antioxidant and antimicrobial functionality for advanced active packaging applications.

### 5.4. Release and Migration Performance

The multifunctional performance of CGA-incorporated packaging systems arises from the interplay between molecular interactions, matrix structure, and environmental responsiveness. Beyond intrinsic antioxidant and antimicrobial properties, the effectiveness of these systems is governed by how CGA is incorporated, mobilized, and activated within the polymer network. Accordingly, this section integrates structure–function relationships with release behavior and activation mechanisms to provide a unified framework for understanding CGA-based packaging performance.

The preservation efficacy of CGA-incorporated packaging ultimately depends on its release and migration behavior. To maintain bioactivity throughout storage, CGA must remain available at an effective concentration in the food-contact environment. However, CGA release is not an intrinsic property of the molecule alone; rather, it is governed by the thermodynamic compatibility between the polymer matrix and the surrounding medium, together with the swelling–relaxation kinetics of the polymer chains.

Vidal, Santos [85] demonstrated this solvent dependency in carboxymethyl cellulose films. In aqueous environments, the hydrophilic matrix swelled readily, and chain relaxation promoted the release of nearly 95% of the loaded CGA within seven days. In contrast, ethanolic simulants, which mimic fatty foods, induced network contraction and restricted chain mobility, limiting CGA release to approximately 39%. These findings highlight a fundamental limitation of simple hydrophilic matrices: they often exhibit pronounced burst release in high-moisture environments, leading to premature depletion of the active agent.

A recurring challenge across fabrication strategies is the initial burst effect, which compromises long-term functionality. In electrospun gelatin/gliadin nanofibers, a substantial fraction of CGA is released within the first few hours [70], largely due to surface-localized CGA formed during rapid solvent evaporation rather than true encapsulation within the fiber core.

To address this limitation, structural engineering strategies have been widely adopted. Multilayer architectures, such as sandwich-structured films, confine CGA within internal layers and delay solvent penetration, thereby extending diffusion pathways [77]. Similarly, nanocarrier-based systems, including halloysite nanotubes, introduce a “labyrinth effect” that increases diffusion-path tortuosity and enables more sustained release profiles [65].

The chemical state of CGA further dictates its functional availability. In chitosan-based systems, covalently grafted CGA has been shown to exhibit higher phenolic accessibility and improved antioxidant performance compared to physically blended systems [61]. This difference arises because physical incorporation can lead to strong electrostatic and hydrogen-bond interactions that restrict molecular mobility, whereas covalent grafting positions CGA in a more accessible or hydrolyzable configuration. Collectively, these findings indicate that advanced CGA-based materials should be designed to regulate not only diffusion pathways but also molecular accessibility within the matrix.

#### 5.4.1. Mechanistic Classification: Diffusion-Controlled Transport vs. Stimuli-Triggered Activation

Building upon the physicochemical principles governing CGA migration, a mechanistic distinction can be made between systems in which functionality depends on passive molecular transport and those in which it is activated by external stimuli. CGA-based films can be categorized into two overarching paradigms: diffusion-controlled systems and stimuli-triggered systems, with conjugation-based strategies representing an intermediate availability-controlled approach.

##### Diffusion-Controlled Systems: Matrix-Regulated Transport

In diffusion-controlled systems, functional performance is directly linked to the migration of CGA from the polymer matrix into the surrounding environment. As discussed in Section 5.4, this process is governed by matrix composition, swelling behavior, and diffusion-path tortuosity.

Structural modifications in these systems primarily aim to regulate transport kinetics. For instance, nanostructured fillers such as layered double hydroxides and halloysite nanotubes act as physical barriers and reservoirs, reducing burst release and prolonging CGA availability [65]. Similarly, multilayer architectures extend diffusion pathways and delay solvent penetration, thereby stabilizing release profiles [77].

Despite these improvements, diffusion-controlled systems remain inherently dependent on environmental conditions, particularly moisture and solvent composition, and therefore provide limited temporal control over functional delivery.

##### Availability-Controlled Systems via Molecular Conjugation

Within diffusion-regulated platforms, molecular conjugation represents a distinct strategy that shifts the focus from mass transport to functional accessibility. In these systems, CGA is covalently bonded to the polymer backbone, effectively restricting molecular mobility at the source.

As demonstrated in chitosan–CGA conjugates [61], this immobilization minimizes uncontrolled migration while preserving bioactivity through surface-accessible phenolic groups or gradual bond cleavage. Compared to physically incorporated systems, where strong intermolecular interactions may hinder release, conjugation provides a more stable and predictable mode of functional expression.

Accordingly, these systems are best described as availability-controlled platforms, in which performance is governed by molecular accessibility rather than bulk diffusion.

##### Stimuli-Triggered Systems: Decoupling Functionality from Migration

In contrast to diffusion-mediated systems, stimuli-triggered platforms decouple functional performance from CGA release. In these systems, CGA remains structurally integrated within the material and contributes to activity through catalytic, photochemical, or interfacial mechanisms activated on demand.

Light-responsive systems exemplify this paradigm. Under irradiation, CGA-containing matrices can generate reactive oxygen species (ROS), leading to antimicrobial activity that is activated only under specific conditions [62]. Similarly, nanozyme-based systems such as CaO_2_@CGA–Fe exploit CGA as a coordinating ligand within catalytic architectures, enabling in situ ROS generation that is further enhanced under near-infrared (NIR) stimulation [16]. Photothermal systems, including Cu–CGA nanoparticles, further demonstrate how localized heating can induce microbial inactivation without relying on CGA migration [66].

A defining feature of these systems is that functional activation is externally controlled, enabling improved spatiotemporal precision, reduced additive migration, and enhanced long-term stability.

##### Design Implications and Trade-Offs

The distinction between diffusion-controlled, availability-controlled, and stimuli-triggered systems has important implications for material design. Diffusion-based platforms are suitable for applications requiring continuous and sustained bioactive release, whereas stimuli-responsive systems enable targeted, on-demand activation with minimal migration.

As reflected in Table 3, improvements in mechanical strength and barrier properties are often associated with reduced CGA mobility, highlighting an inherent trade-off between structural integrity and functional delivery. Therefore, the rational design of CGA-based films requires aligning matrix architecture, molecular state, and activation mechanism with the intended application scenario, balancing the need for a robust physical barrier with the kinetic requirements of bioactive functionality.

## 6. Applications of CGA-Incorporated Active and Intelligent Films

While optimizing mechanical and barrier integrity is essential, the distinguishing feature of CGA-incorporated films lies in their active biological capabilities. CGA serves as a functional agent whose bioactivity is closely linked to its release kinetics and interaction with the polymer matrix. The application of CGA has evolved from simple physical blending to sophisticated structural engineering. It is now a versatile bioactive agent addressing diverse preservation challenges (Table 4).

In protein-rich and lipid-sensitive products, such as shrimp and fish, CGA effectively retards melanosis and inhibits spoilage organisms. Its performance depends heavily on the incorporation strategy. For instance, chemical conjugation offers sustained antioxidant reservoirs. In contrast, physical incorporation facilitates immediate release for rapid bacterial membrane disruption [61,70,84].

Beyond antimicrobial action, CGA plays a critical structural role in fruit preservation. In biopolymer matrices like silk fibroin and starch, it acts as a cross-linker. By promoting β-sheet formation or reinforcing hydrogen bonding through high-shear homogenization, CGA densifies the film structure. This significantly reduces water vapor permeability and extends the shelf-life of moisture-sensitive fruits like strawberries [59,72].

Recent studies underscore the necessity of using multi-component matrices to maximize these benefits. For example, in a ternary starch-based system crosslinked with citric acid, CGA yielded profound synergistic effects for preserving fresh-cut cherry tomatoes [60]. This formulation used the densified network to suppress fruit respiration and transpiration. Simultaneously, the sustained release of CGA provided robust antioxidant and antimicrobial actions. As a result, tomatoes packaged in these films maintained a firmness of 7.64 N after 7 days, compared to only 2.08 N in unpackaged controls.

CGA-incorporated matrices are also highly effective for transpirative produce like oyster mushrooms (*Pleurotus geesteranus*) [21]. These mushrooms are susceptible to severe moisture loss due to their lack of a protective epidermal layer. A recent study showed that a chitosan-based film reinforced with CGA-loaded nanoparticles fundamentally altered their spoilage kinetics. Over 10 days at 4 °C, the CGA-composite film restricted weight loss to only 14.76%, compared to 87.53% in controls. This was primarily driven by an active biochemical mechanism. CGA effectively scavenged the free radicals generated during the mushrooms’ intense respiratory burst. This membrane-stabilizing effect, coupled with CGA’s antimicrobial ability, prevented tissue softening and off-odor development.

Direct application of CGA as an active coating on perishable fruits, such as strawberries, reveals its capacity to reprogram postharvest physiology [86]. CGA application fundamentally alters the senescence trajectory by suppressing both respiratory rates and ethylene emission. This metabolic attenuation is characterized by a reduction in membrane lipid peroxidation. This is evidenced by the suppression of malondialdehyde (MDA) accumulation and minimized electrolyte leakage (EL). By mitigating oxidative stress, CGA preserves cellular turgor and delays the enzymatic degradation of anthocyanins. These interventions suggest that CGA might stimulate alternative energy pathways, such as the GABA shunt, helping the tissue maintain structural integrity.

Finally, recent advancements have transitioned CGA into stimuli-responsive and “on-demand” intelligent systems. This is exemplified by photothermal and photodynamic films. In these systems, CGA works with metal ions or photosensitizers to generate localized hyperthermia or reactive oxygen species (ROS) upon light irradiation. This achieves rapid surface sterilization for commodities like cherry tomatoes [16,62,66].

A recent breakthrough utilized CGA co-polymerized with allicin to synthesize negatively charged nanoparticles. These were used to adsorb a cationic porphyrin photosensitizer [87]. When incorporated into a CMCS/SA matrix, this composite exhibited potent photosensitivity. Upon exposure to visible light, the system generated massive amounts of ROS, achieving 100% antibacterial efficacy. Furthermore, the coating provided >80% UV shielding while maintaining >85% transparency. It was also easily washable, making it consumer-friendly. Additionally, CGA can be converted into carbon nanodots to create fluorescent sensors for monitoring spoilage indicators [18]. Collectively, these studies illustrate a paradigm shift where CGA is now a functional building block for next-generation, multi-modal food packaging systems.

**Table 4:** Application of CGA-incorporated films in food preservation.

Active Packaging System	Preparation Method	Packaged Food	Findings	Ref.
**Fruits**				
CGA-derived carbon dots/alginate film	Solvent casting	Fresh-cut apples	Effective inhibition of microbial growth and enzymatic browning	[74]
Starch-based active films by citric acid and CGA	Solvent casting	Fresh-cut cherry tomato	Exhibited synergistic gas and water vapor barrier properties, suppressing fruit respiration and transpiration. Effectively delayed enzymatic browning and microbial colonization while significantly retaining tissue firmness (7.64 N vs. 2.08 N in control) over 7 days of storage.	[60]
Chitosan film containing CaO_2_/CGA/Fe nanoparticles	Solvent casting	Cherry tomatoes	Increased the shelf-life by up to 12 days and the physicochemical indices (weight loss, hardness changes, pH value, soluble solids, titratable acid content, and total phenolic content) retained during storage.	[16]
Carrageenan/gelatin film containing Cu-CGA nanoparticles	Solvent casting	Cherry tomatoes	Films reduced weight loss to 16.63% (vs. 31.84% in control), best preserved firmness, and minimized total soluble solids decline over 18 days, effectively extending cherry tomato shelf life.	[66]
CGA–polyurethane film	Solvent casting	Grape	Compared with the control (14% weight loss), the film limited mass loss to 2%. Color stability was markedly improved, with visibly reduced browning. The film also exhibited the slowest hardness loss. Metabolic parameters were stabilized, as reflected by a much slower increase in pH and the retention of total soluble solids near their initial levels after 8 days.	[80]
Pullulan/gelatin film containing CGA/chitosan nanoparticles	Solvent casting	Banana (coating) and chicken (packaging film)	For banana: Delayed browning and moisture loss; maintained firmness and appearance quality up to 5–7 days at 25 °C; improved consumer acceptability by limiting oxygen ingress and microbial contamination. For chicken: Reduced total bacterial counts and bacterial diversity during storage; broad-spectrum antibacterial activity of CGA–chitosan films effectively prolonged the shelf life of fresh chicken.	[79]
CGA–carbon nanodots poly(vinyl alcohol) film	Solvent casting	Banana	Reduced fruit decay and delayed ripening via antioxidant activity; enabled rapid fluorescence-based detection of alkaline spoilage and Al^3+^ residues (<100 mg kg^−1^) → combined active preservation and intelligent sensing functionality.	[18]
		Deep-fried dough sticks, spoiled milk, and preserved eggs	Showing difference fluorescence for each product under 365 nm UV light.	
CGA–sweet whey/starch film	Solvent casting	Banana (coating)	Coatings significantly delayed browning and maintained firmness of bananas, extending shelf life; 1% CGA was sufficient, with no added benefit at higher loading.	[55]
CGA–agar film	Solvent casting	Cherry	films reduced decay (75% → 12.5%), limited weight loss (~42% in control), preserved color (ΔE ≈ constant), delayed softening, restrained TSS increase, and minimized pH rise, extending cherry shelf life to 9 days at room temperature.	[62]
Chitosan film loaded with CGA–chitosan oligosaccharide nanoparticles	Solvent casting	*Pleurotus geesteranus*	Drastically restricted weight loss (14.76% vs. 87.53% in control) over 10 days. Scavenged respiratory free radicals and delayed membrane lipid peroxidation, effectively maintaining cellular integrity and preventing tissue softening, pileus whitening, and off-odors.	[21]
CGA–allicin–porphyrin–carboxymethyl chitosan/sodium alginate film	Solvent casting	Perishable fruit model (coating)	Demonstrated potent photodynamic antimicrobial activity (100% efficiency) via ROS generation under visible light. The smart coating provided >80% UV-shielding, high transparency (>85%), and easy washability, effectively extending the shelf-life of the preserved fruits.	[87]
CGA–corn starch film	Solvent casting	Strawberry	Film reduced browning (BI: 1.67 vs. 4.00), lowered weight loss (~28%), preserved firmness, and maintained phenolics and flavonoids over 7 d, demonstrating effective moisture and oxidative protection.	[59]
CGA–corn starch/polyvinyl alcohol film	Solvent casting	Strawberry	Markedly improved quality: weight loss was reduced by ~65%, accompanied by enhanced retention of ascorbic acid (~16%), total soluble solids (~3%), titratable acidity (+0.13 g/100 g), and firmness (~2 N). Microbial growth was strongly suppressed, sensory acceptability remained high, and strawberry shelf life was extended to at least 7 days.	[81]
-	-	Strawberry (coating)	Acted as a potent metabolic modulator by significantly suppressing respiratory rates and ethylene emission. Mitigated intracellular oxidative stress (reduced MDA and electrolyte leakage), preserving cellular turgor, titratable acidity, and delaying anthocyanin degradation.	[86]
CGA–cellulose nanocrystal/silk-based film	Solvent casting	Strawberry	The films maintained brighter skin color (↓ ΔE), reduced weight loss by ~50%, slowed firmness degradation, and decreased decay percentage by ~70%. They also delayed pH increase, mitigated titratable acidity loss, retained total soluble solids, and preserved total phenolic content during storage → extending strawberry shelf life to 15 days.	[72]
**Protein foods**				
chitosan films with conjugated (CGA-g-CS) or incorporated CGA	Solvent casting	Shrimp	CGA-g-CS coating most effectively preserved shrimp quality during 8 days of storage by markedly reducing weight loss (3.5% vs. 13.2% in control), suppressing pH increase, delaying TVB-N accumulation (18.17 vs. 30.27 mg/100 g), lowering total bacterial counts (4.89 vs. 6.70 log CFU/g), and maintaining acceptable sensory quality. Superior performance was attributed to reduced water vapor permeability and enhanced antioxidant and antibacterial activity via CGA–chitosan conjugation.	[61]
CGA–gelatin/wheat gliadin electrospun film	Electrospinning	Grass carp fillets	Compared to the control, weight loss and TVC were reduced, while texture firmness was better maintained; TVB-N, pH, and TBARS showed significant decreases during 10 days of storage.	[70]
CGA–corn starch emulsion coating	Emulsification	Cod fillet	After 12 days of storage, the CGA-coated samples showed reductions of 20.8% in TVB-N, 14.6% in TVC, 41.1% in TBARS, and 3.5% in pH compared to the control. The emulsion extended shelf life from 6 to 8 days.	[84]
Polyvinyl alcohol film loaded with CGA/Walnut isolate protein–propylene glycol alginate nanoparticles	Solvent casting	Pork fillet	Films maintained higher a* values, reduced b* values, and limited weight loss (0.126% after 8 d). Spoilage indicators were suppressed, with TVB-N reduced from 23.64 to 14.58 mg/100 g and TVC maintained below 7 log CFU/g. Encapsulation of CGA in WNPI/PGA enabled sustained release, yielding superior antioxidant, antimicrobial, and texture-preserving performance.	[7]
CGA–carboxymethyl cellulose films	Solvent casting	Fish oil	Peroxide value (PV) and thiobarbituric acid reactive substances (TBARS) decreased by ˃80% and ˃76%, respectively.	[85]
CGA–chitosan coating	Mixing	Snakehead fish	Antimicrobial activity did not differ significantly; however, color, antioxidant capacity, and pH showed significant differences. Lipid and protein oxidation were effectively inhibited in fillets. All coatings delayed pH increase and reduced browning.	[58]
CGA–gelatin conjugate	Conjugation	Fish (coating)	Coating markedly suppressed lipid oxidation; after 48 h, TBARS values remained <9 mg MDA/L oil compared with complete oxidation in the control. It showed superior antioxidant efficacy over free CGA at equal loading.	[71]

## 7. Current Challenges, Emerging Solutions, and Future Perspectives

**Figure 4:**
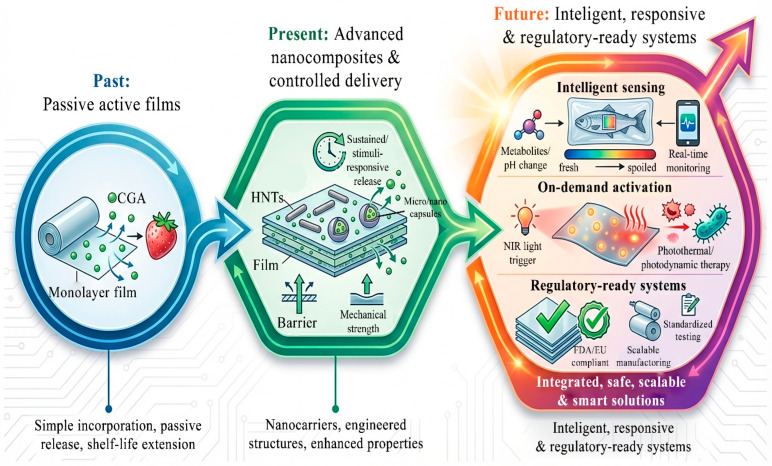
Future perspectives, critical challenges, and prospective advancements in the scale-up, regulatory compliance, and commercial translation of CGA-incorporated packaging technologies.

Despite the demonstrated multifunctionality of CGA in active and intelligent films, several scientific, technological, economic, and regulatory challenges must be addressed before large-scale industrial translation can be realized (Figure 4). This section critically outlines the key challenges, summarizes emerging mitigation strategies, and highlights future research directions.

### 7.1. Key Challenges Limiting CGA-Incorporated Films

#### 7.1.1. Chemical Instability and Sensitivity: The “Achilles’ Heel”

A primary limitation of CGA is its inherent susceptibility to oxidation, hydrolysis, and pH-induced degradation. Owing to the presence of an ester linkage and vicinal hydroxyl groups in its catechol moiety, CGA is particularly vulnerable to alkaline conditions and thermal processing. During film extrusion or prolonged storage, CGA can oxidize into quinone derivatives, causing undesirable browning and a concomitant loss of bioactivity. As reported by Ren, Fan [84], the instability of free CGA significantly restricts its direct use in packaging systems.

#### 7.1.2. Uncontrolled Release Kinetics and the “Burst Effect”

Most CGA-incorporated films are fabricated via solvent casting, where CGA is physically entrapped within hydrophilic polymer matrices. This often results in a rapid initial migration (“burst release”), followed by premature depletion of the active compound. Consequently, antioxidant and antimicrobial protection diminishes during the later stages of storage, which is particularly problematic for foods requiring extended shelf life.

#### 7.1.3. Organoleptic Impact and Consumer Acceptance

CGA presents sensory challenges related to its intrinsic color and bitterness, especially upon oxidation. Increasing CGA content has been shown to reduce film lightness (L*) and increase yellowness (b*), potentially impairing product visibility despite improving UV-shielding performance [62]. Moreover, migration of CGA at high concentrations may adversely affect the flavor of delicately flavored foods. Notably, systematic sensory evaluations remain largely absent from current literature.

#### 7.1.4. Economic Feasibility and Scalability

A substantial gap persists between laboratory-scale fabrication methods (predominantly solvent casting) and industrial processing techniques such as extrusion or blow molding. In addition, the relatively high cost of purified CGA limits its feasibility for commodity packaging applications.

#### 7.1.5. Regulatory Uncertainty and Safety Considerations

While CGA is generally recognized as safe (GRAS) for direct oral consumption, its application in food-contact materials (FCMs) is subject to more stringent regulatory requirements. Under frameworks such as EU Regulation No. 10/2011 and the FDA’s Food Contact Substance (FCS) notification system, any substance intended to be used in active packaging must undergo comprehensive safety evaluation, particularly regarding its potential migration into food [88].

In this context, both Overall Migration Limits (OML) and Specific Migration Limits (SML) must be established to ensure that the release of CGA or its potential degradation products (e.g., quinone derivatives) remains within acceptable safety thresholds and does not adversely affect the organoleptic properties of food [89].

However, available data on the long-term toxicological effects of CGA-derived oxidation products are still limited, highlighting the need for further investigation. Therefore, a detailed understanding of migration behavior and kinetics under different food simulants (e.g., aqueous, acidic, and alcoholic media) is essential for the safe and effective implementation of CGA-based active packaging systems.

### 7.2. Emerging Solutions and Mitigation Strategies

To address chemical instability and burst release, recent studies have shifted from simple physical blending toward protective engineering strategies. These include chemical conjugation (e.g., CGA–chitosan grafting) and encapsulation within nanocarriers such as halloysite nanotubes, core–shell emulsions, or metal–phenolic networks, which shield CGA from premature degradation and enable sustained release [61,65].

Stimuli-responsive systems represent another promising solution. Triggered release mechanisms activated by temperature, light, pH, or humidity—such as near-infrared photothermal activation or nanozyme-based catalytic platforms—allow CGA activity to be synchronized with food spoilage dynamics, thereby extending functional lifespan [16,66].

From an economic perspective, valorization of agricultural by-products rich in CGA (e.g., coffee silverskin, green coffee oil residues, potato peels) has emerged as a viable strategy to reduce raw material costs while aligning CGA-incorporated films with circular economy principles [85].

### 7.3. Future Perspectives: Toward Intelligent CGA-Incorporated Films

Beyond conventional active films, a critical yet underexplored frontier lies in CGA-enabled intelligent films. While most studies have focused on CGA as an antioxidant or antimicrobial agent, its intrinsic optical, redox, and pH-responsive properties position it as an excellent candidate for sensing and signaling applications. CGA-derived fluorescent platforms, and carbon nanodot systems have already demonstrated feasibility for real-time freshness monitoring and spoilage detection, but remain an emerging niche in the literature.

Future research should therefore prioritize the integration of CGA into multifunctional intelligent packaging architectures that combine preservation, sensing, and on-demand responsiveness, while maintaining mechanical integrity and consumer acceptability. Advancing such systems—alongside robust sensory evaluation, scalable processing routes, and regulatory validation—will be essential for transitioning CGA-containing packaging systems from laboratory prototypes to commercially viable intelligent food packaging solutions.

## 8. Conclusions

This review demonstrates that CGA functions far beyond a conventional antioxidant additive. It acts as a multifunctional modulator that simultaneously influences the structural organization and functional performance of biopolymer-based packaging. The evidence synthesized here shows that CGA’s molecular versatility—specifically its ability to participate in hydrogen bonding, electrostatic interactions, and covalent conjugation—enables it to tailor mechanical strength, barrier properties, and optical behavior. Importantly, the performance of these systems is not intrinsic. Instead, it is highly dependent on the incorporation strategy, matrix composition, and microstructural design.

A key insight from this analysis is the ongoing transition from passive packaging toward responsive and intelligent systems. While recent studies demonstrate the feasibility of CGA-enabled platforms with sensing capabilities, research remains heavily skewed toward active packaging. The limited integration of CGA into real-time sensing and signal transduction highlights a critical gap. There is a clear disconnect between its molecular potential and its practical deployment in intelligent technologies.

Several challenges continue to constrain the translation of these systems into real-world applications. These include the chemical instability of CGA during processing, uncontrolled burst-release behavior in hydrophilic matrices, and an incomplete understanding of long-term performance in complex food environments. Furthermore, gaps in regulatory frameworks, including insufficient migration data and toxicological evaluations of degradation products, limit commercialization.

Future research should prioritize integrated strategies to stabilize CGA and control its release kinetics. Approaches such as chemical conjugation, core–shell encapsulation, and advanced nanostructuring offer promising routes. Furthermore, expanding CGA applications into intelligent systems—particularly for real-time sensing and responsive functionality—is essential. Finally, addressing scalability through the utilization of agro-industrial byproducts and establishing clear regulatory pathways will be critical. These steps will enable the transition from laboratory-scale innovations to commercially viable and sustainable packaging solutions.

## Data Availability

No data was used for the research described in the article.
